# Proteometabolomic Analysis Reveals Molecular Features Associated with Grain Size and Antioxidant Properties amongst Chickpea (*Cicer arietinum* L.) Seeds Genotypes

**DOI:** 10.3390/antiox11101850

**Published:** 2022-09-20

**Authors:** Francisco R. Quiroz-Figueroa, Juan L. Monribot-Villanueva, Esaú Bojórquez-Velázquez, Rosa L. Gómez-Peraza, José M. Elizalde-Contreras, Mirna V. Bautista-Valle, José A. Guerrero-Analco, Maribel Valdez-Morales, Rupesh Kumar Singh, Eliel Ruiz-May

**Affiliations:** 1 Laboratorio de Fitomejoramiento Molecular, Centro Interdisciplinario de Investigación para el Desarrollo Integral Regional-Unidad Sinaloa, Instituto Politécnico Nacional, Boulevard Juan de Dios Bátiz Paredes # 250, Col. San Joachin, Guasave 81101, Sinaloa, Mexico; 2 Red de Estudios Moleculares Avanzados, Instituto de Ecología A. C., Cluster BioMimic®, Carretera Antigua a Coatepec 351, Congregación el Haya, Xalapa 91073, Veracruz, Mexico; 3 Centre of Molecular and Environmental Biology, Department of Biology, University of Minho, Campus of Gualtar, 4710-057 Braga, Portugal or; 4 InnovPlantProtect Collaborative Laboratory, Department of Protection of Specific Crops, 7350-999 Elvas, Portugal

**Keywords:** stress-relate proteins, polyphenols, isobaric labeling, multiple reaction monitoring

## Abstract

Legumes are an essential source of nutrients that complement energy and protein requirements in the human diet. They also contribute to the intake of bioactive compounds such as polyphenols, whose content can vary depending on cultivars and genotypes. We conducted a comparative proteomics and metabolomics study to determine if there were significant variations in relevant nutraceutical compounds in the five genotypes of Kabuli-type chickpea grains. We performed an isobaric tandem mass tag (TMT) couple to synchronous precursor selection (SPS)-MS3 method along with a targeted and untargeted metabolomics approach based on accurate mass spectrometry. We observed an association between the overproduction of proteins involved in starch, lipid, and amino acid metabolism with gibberellin accumulation in large grains. In contrast, we visualized the over-accumulation of proteins associated with water deprivation in small grains. It was possible to visualize in small grains the over-accumulation of some phenolics such as vanillin, salicylic acid, protocatechuic acid, 4-coumaric acid, 4-hydroxybenzoic acid, vanillic acid, ferulic acid, and kaempferol 3-*O*-glucoside as well as the amino acid l-phenylalanine. The activated phenolic pathway was associated with the higher antioxidant capacity of small grains. Small grains consumption could be advantageous due to their nutraceutical properties.

## 1. Introduction

Legumes have been incorporated as the second most important vegetable food source after cereals. Their main nutritional value lies in their protein content of 20–50%, in addition to complementing the essential amino acid scheme in grain-based diets [1]. Chickpeas (*Cicer arietinum* L.), also known as garbanzo beans, are globally the third most produced and second most consumed food legume [2]. Its grains are the main consumable part of the plant. They have high protein content with essential amino acids, unsaturated fats, vitamins, carbohydrates, crude fiber, minerals, and carotenoids that meet the nutritional need of both humans and animals [2,3,4,5,6,7]. India is the leading producer of chickpea grain, with a significant portion of the almost 12 million tons of annual global production [8]. Developing countries like Mexico, Malawi, Morocco, and Syria significantly contribute to the international chickpea commercialization [8]. In Mexico, during the agricultural cycle 2020–2021, ca 92,000 ha were designated to plant chickpeas with a total yield of 172,000 t (USD129 million). The Sinaloa state is the leading producer of chickpeas, with more than 70% destined for export [9].

Chickpea grains are classified into two types based on color, size, and shape: Desi and Kabuli. Desi is a darker-colored (mostly brown) and angular-shaped seed with a prominent beak, while Kabuli is white or cream-colored, rounder in shape, and has a small beak [10]. The Kabuli type is used for human consumption in a variety of meals. Classification based on seed size is an important trait for consumer preference and trade [10]. Additionally, seed size has been described as a factor in plant adaptation and fitness, making it an important research area in developmental biology [11]. One of the most important commercial traits is seed size, which correlates positively with seed weight. Researchers and plant breeders know the importance of large size seeds and seed weight in the yield and production of food crops. Planting large seeds has been shown to produce higher seed yield and larger seeds for commercialization or consumption [12]. Besides, the nutraceutical content related to polyphenols has been evaluated in chickpeas and other legumes, focusing on evaluating the bio-accessibility depending on the cooking process or hemagglutinating capacity [13,14]. However, the limited effort in massive profiling of nutraceutical molecules in chickpeas is worth noting. For example, a previous study suggested different contents of total flavonoids and polyphenols between Desi and Kabuli chickpeas, where it was possible to determine up to 11- and 13-times higher flavonoids and polyphenols in the colored seed genotypes than cream and beige color seeds [15]. However, the identification of polyphenol-based metabolomics tools in these total extracts has not been carried out in chickpeas. Additionally, various germination conditions like dark or light and the presence of ethanol or salts influence chickpea content of essential isoflavone-like compounds; applying light is the better treatment to increase the isoflavone content in a shorter time. For example, formononetin and biochanin A increase from 0.1 and 0.18 to 1.54 and 2.34 mg/g, respectively, after eight days under this condition [16]. These findings suggested an added value to chickpeas as a functional food by rationally incorporating them into diet schemes focused on increasing the content of antioxidant compounds or applied as an additive to enrich these nutrients in more complex food systems such as processed foods.

The grain filling stage defines the seed protein content and may vary among plant families and in different accessions within species. This may depend mainly on the basic metabolic pathways of each plant species. Besides, pioneer studies provided the first insight into the nutraceutical potential of chickpea, mainly anti-inflammatory effects mediated by biochanin A and daidzein, as well as the antioxidant capacity of other phenolic compounds [17]. However, multiomics tools like proteomics and metabolomics should be applied to understand grain size‘s molecular foundation and profile bioactive molecules and their corresponding metabolic pathways. This information will be invaluable for seed researchers in grain breeding programs. Proteometabolomics approaches have been used to identify differentially accumulated proteins and metabolites under diverse conditions [18]. These high-throughput-based systems have been applied in *Medicago truncatula*, *Lotus japonicus*, and soybeans, while chickpeas, lensculinaris, mung beans, and peanuts have been marginalized leguminous crops [19,20,21]. Based on this, the aim of this work was to study the differential proteome and metabolome of five Kabuli-type chickpea genotypes to determine molecular signatures and nutritional traits associated with grain size. Our results indicated a high content of antioxidants in small chickpea seeds, and production and consumption of them may bring health benefits to consumers.

## 2. Materials and Methods

### 2.1. Biological Material

Five commercial Kabuli-type chickpea genotypes (BigF2 and SmallCo from 2017; Blanco Sinaloa BigCo, SmallF1, and Jumbo from 2018) were obtained from local farmers in Sinaloa, Mexico (25.6345 latitude and −108.420443 longitude). The temperatures for the agricultural cycle of 2017–2018 were 11–28 °C in November and 15–18 °C in February, and for the agricultural cycle of 2018–2019 were 21–29 °C in November and 13–23 °C in February (weatherspark.com (accessed on 27 July 2022)). The grains were sieved and freed from impurities, and rinsed with distilled water from previous protein and metabolites extraction.

### 2.2. Seed Measurement

The length, width, and thickness of 25 seeds per genotype were measured using a digital caliper (Model 3415 Traceble^®^, Friendswood, TX, USA). The seed weight (*n* = 25 per phenotype) was determined using an analytical balance (Model PW254 ADAM, Milton Keynes, UK).

### 2.3. Proximate Analysis

The proximate composition of the chickpea seeds was determined according to the Association of Analytical Communities (AOAC International, 1995) methods for ash (Section 942.05), crude fiber (AOAC Section 978.10), and moisture (AOAC Section 930.15). Crude protein was determined by the micro-Kjeldahl method (AOAC Section 976.05), and crude lipids were determined by the AOAC method (AOAC Section 920.39).

### 2.4. Total Antioxidant Capacity by 2,2-Diphenyl-1-picrylhydrazyl (DPPH) and Oxygen Radical Absorbance Capacity (ORAC)

The crude methanolic extract was obtained from 200 mg of sample (fine flour) suspended in 8 mL of methanol (ACS, Tedia, CRT-Mexico). The mixture was placed into 50 mL centrifuge tubes and sonicated for 30 min (FS20, Fisher Scientific, Waltham, MA, USA), during which they were mixed and vortexed (VX100, Labnet, Madrid, Spain) every 10 min. Then, the samples were incubated (VWR Avantor, Radnor, PA, USA) for 15 h (25 °C/darkness, 200 rpm) and centrifuged at 4000 rpm/10 min in a Hermle Z 366 centrifuge (Labortechnik GmbH, Wehingen, Germany). The supernatant was decanted and stored at −20 °C, and the pellet was re-extracted for 2 h and processed again as described before. The supernatants from the two extractions were combined and concentrated to dryness in a rotary evaporator under reduced pressure (Yamato, Santa Clara, CA, USA) at 37 °C and reconstituted in 1 mL of methanol (ACS, Tedia, CTR-Mexico). The antioxidant capacity of the methanolic extracts was determined by the DPPH and ORAC methods as described by Cardador-Martínez, et al. [22] and Prior et al. [23], respectively. The results were expressed as µmol equivalents of Trolox/g of flour.

### 2.5. Proteomic Analysis

Proteomic analysis, including protein extraction, digestions, peptide fractionation, TMT labeling, SPS-MS3, and data analysis were conducted as described by Monribot-Villanueva et al. [24,25]. Details of the proteomic analysis are presented in Appendix A. We considered two biological replicates for each grain and carried out a 10plex TMT tag according to the manufacturer’s instructions as follows: for SmallF1, we used 126 and 127N; for BigF2 we used 127C and 128N; for SmallCo we used 128C and 129N, for BigCo we used 129C and 130N, and for Jumbo we used 130C and 131. For protein identification, we used the chickpea UniProt Reference proteome (UP000087171) as the database. Data are available via ProteomeXchange with identifier PXD030279.

### 2.6. Metabolomic Analysis

Untargeted metabolomic analyses of methanolic extracts were performed as indicated by Monribot-Villanueva, et al. [24,25] in an Ultra-High Performance Liquid Chromatograph coupled to a High-Resolution Mass Spectrometer (UPLC-HRMS-QTOF; Class I-Synapt G2-Si, Waters, Milford, MA, USA). Metabolomics data were first processed with the Masslynx and Markerlynx softwares (versions 4.1, Waters, Milford, MA, USA). Tentative identification was performed using two approaches considering a maximum mass error of ±5 ppm. The first approach was by the MetaboAnalyst bioinformatic platform (https://www.metaboanalyst.ca/MetaboAnalyst/home.xhtml, last accessed on 1 July 2022) with the Peaks to Pathways tool using the algorithm Mummichog and the rice reference metabolome. The second strategy was a manual annotation with *m*/*z* values using the public database Foodb (https://foodb.ca/, last accessed on 1 July 2022). The identification and quantification of phenolic compounds were based on dynamic multiple reaction monitoring methods as was previously reported in Juárez-Trujillo et al. [26], Camacho-Vázquez et al. [27], and Monribot et al. [21,22]. Detailed information is shown in Appendix A. Phenolics heatmap was performed with MetaboAnalyst bioinformatic platform [28].

### 2.7. Statistical Analysis

Raw data were analyzed to determine whether they differed from a normal distribution (Shapiro-Wilk test). The homoscedasticity of the data was tested using the Barlett test for normally distributed data and the Fligner-Killeen test for data with non-normal distribution. Variables that conformed to parametric assumptions were analyzed using one-way analysis of variance (ANOVA) and the Duncan Means Test (α < 0.01); those that did not were analyzed using the non-parametric Kuskal-Wallis test and pairwise Wilcox test. We also generated a correlation coefficient matrix between protein content and the morphometric variables, among morphometric variables, and among proximate composition variables. All statistical analysis was carried out in the R environment. Origin version 8.5.1 (OriginLab, Nothampton, MA, USA) and CorelDraw version 17.1.0.572 (Corel, Ottawa, Canada) were used to generate graphs and figures, respectively.

## 3. Results

### 3.1. Phenotypic Features of Chickpea Genotypes with Different Grain Size

The five genotypes of Kabuli chickpeas studied shared phenotypic characteristics. They were white-cream colored, pronounced rugosity, rounded, and with a small beak (Figure S1A). The theoretical volume for the large grains (BigF2, Jumbo, and BigCo) was between 1100 and 1540 mm^3^ and for the small grains (SmallF1 and SmallCo) was 700–1000 mm^3^ (Figure S1B). The morphometric traits showed statistical differences between the large and small grains in all parameters recorded (Figure S1C). Small grains exhibited comparable physical parameters between them (SmallF1 and SmallCo). Among the three large grains, BigCo had larger weight values than the other two large grains (Figure S1D).

### 3.2. Proximate Analysis of Chickpea with Different Grain Size

The proximate composition of chickpea grains was evaluated among the five genotypes (Table S1). The BigF2 genotype had the highest protein content, followed by Jumbo, Small F1, SmallCo, and BigCo. Lipid content was highest in SmallF1 and lowest in SmallCo and BigF2. The crude fiber content varied from 4.06 to 7.33%; the Jumbo genotype had the highest content, followed by SmallCo, BigF2, BigCo, and SmallF1. The ash content was relatively stable among the five genotypes, ranging from 3.02 to 3.23%, with no statistical differences. The nitrogen-free extract content ranged from 59.66 to 53.62%. The BigCo and SmallCo genotypes had the greatest nitrogen-free extract content, followed by BigF2, Jumbo, and SmallF1. There were no correlations between protein content versus morphometric characteristics such as weight, length, etc., but there were strong positive correlations among the morphometric characteristics (Figure S2A). When considering correlations among proximate variables, there were positive and negative correlations, except for protein content which was not correlated with any other factor (protein vs. moisture, lipids, ash, and crude fiber; Figure S2B). Also, the ash did not correlate with fiber content, nitrogen-free extract content, or moisture (Figure S2B).

### 3.3. Comparative Proteomics Revealed Contrasting Proteome Profile in Small vs. Large Grain Genotypes

**Figure 1:**
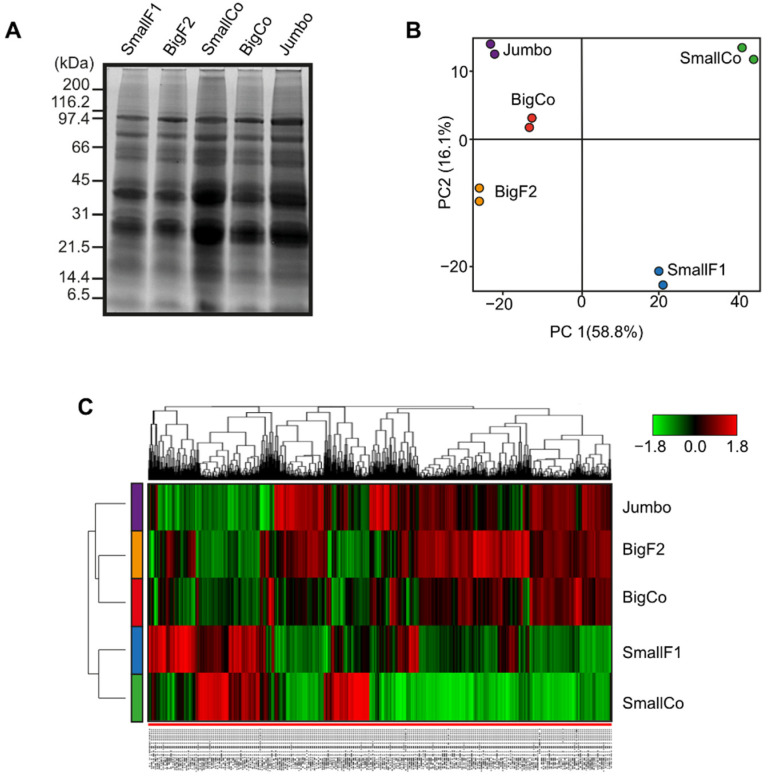
Comparative proteomic analysis of chickpea grain genotypes that differ in size. (**A**) Grain protein pattern visualized in an SDS-PAGE; protein standard ladder is shown on the left side of the gel. (**B**) Principal component analysis and (**C**) heatmap based on protein abundance. SmallF1 = small farmer 1; BigF2 = big farmer 2; SmallCo = small commercial; BigCo = large commercial.

Protein bands in SDS-PAGE had similar patterns in all chickpea genotypes analyzed (Figure 1A). There were prominent protein bands between 30 and 45 kDa, and there were no visibly noticeable differences among protein profiles. Under comparative proteomics based on TMT labeling and the SPS-MS3 approach, the analytical pipeline identified 1854 proteins associated with 6107 peptide groups and 54,618 MS/MS spectra (ProteomeXchange identifier: PXD030279). The principal component analysis of the identified protein abundances exhibited a grouping pattern. Large grains exhibited similar protein abundances that contrasted with the small grains (Figure 1B). The heatmap based on protein abundance also showed differences among the chickpea genotypes analyzed. Hierarchical clustering clearly separated the small genotypes from the large ones (Figure 1C).

**Figure 2:**
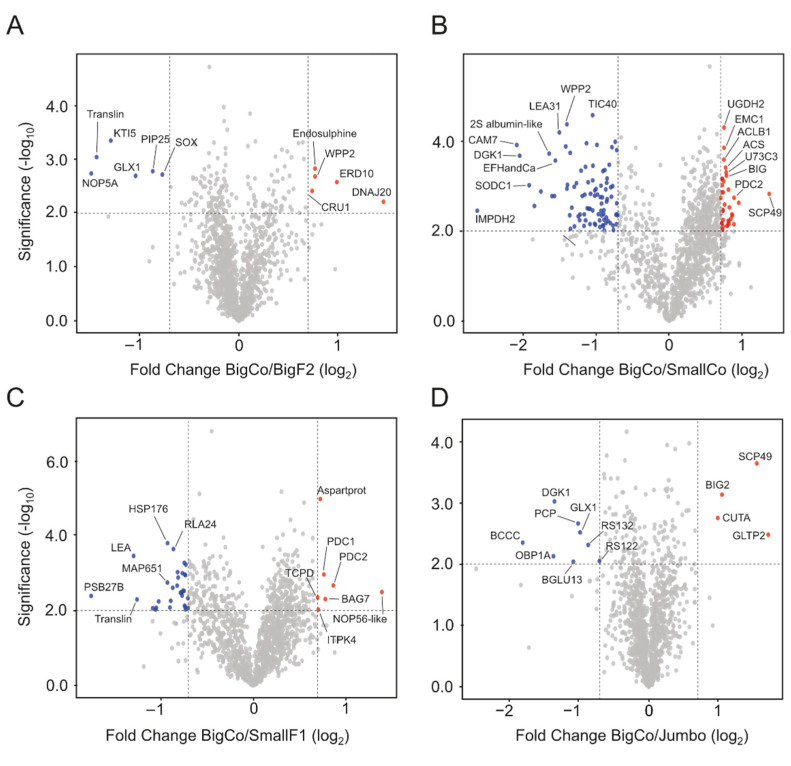
Representation of protein abundances in volcano plots based on different log2 ratios ((**A**), BigCo/BigF2; (**B**), BigCo and SmallCo; (**C**), BigCo and SmallF1; (**D**), BigCo and Jumbo) and significant values (*p*-value −log10). Each figure shows the most significant differential proteins. In (**A**), Kunitz trypsin inhibitor 5 (KTI5), Translin family protein, probable nucleolar protein 5-1 (NOP58), lactoylglutathione lyase 1 (GLX1), probable aquaporin PIP2-5 (PIP25), sulfite oxidase (SOX), chaperone protein (DNAJ20), dehydrin (ERD10), endosulphine (Q93Z49), WPP domain-containing protein 2 (WPP2), and 12S seed storage protein CRA1 (CRU1). In (**B**), calmodulin 7 (CAM7), diacylglycerol kinase 1 (DGK1), inosine-5′-monophosphate dehydrogenase 2 (IMPDH2), late embryogenesis abundant protein 31 (LEA31), WPP2, TIC40, 2S albumin-like (A0A1S2XDF0), calcium-binding EF-hand family protein (EFHandCa), superoxide dismutase Cu-Zn 1 (SODC1), serine carboxypeptidase-like 49 (SCP49), UDP-glucose 6-dehydrogenase 2 (UGDH2), ATP-citrate synthase beta chain protein 1 (ACLB1), ER membrane protein complex subunit 1 (EMC1), acetyl-coenzyme A synthetase (ACS), UDP-glycosyltransferase 73C3 (U73C3), and auxin transport protein (BIG). In (**C**), photosystem II D1 precursor processing protein (PSB27B), LEA, 17.6 kDa class II heat shock protein (HSP176), 60S acidic ribosomal protein P2-4 (RLA24), translin (A0A178W0K0), 65-kDa microtubule-associated protein 1 (MAP651), eukaryotic aspartyl protease family protein (Aspartprot), NOP56-like pre RNA processing ribonucleoprotein (NOP56-like), pyruvate decarboxylase 1 (PDC1), PDC2, T-complex protein 1 subunit δ Chaperonin CCT4 (TCPD), inositol 1,3,4-trisphosphate 5/6-kinase 4 (ITPK4), and BAG family molecular chaperone regulator 7 (BAG7). In (**D**), serine carboxypeptidase-like 49 (SCP49), brefeldin A-inhibited guanine nucleotide-exchange protein 2 (BIG2), Protein CutA (CUTA), glycolipid transfer protein 2 (GLTP2), diacylglycerol kinase 1 (DGK1), biotin carboxylase (BCCC), period circadian protein (PCP), oil body-associated protein 1A (OBP1A), lactoylglutathione lyase 1 (GLX1), 40S ribosomal protein S13-2 (RS132), 40S ribosomal protein S12-2 (RS122), and β-glucosidase 13 (BGLU13).

To analyze differential proteins, we considered a fold change value of 1.5 (log2 fold change value: 0.58) in comparison with genotypes, including BigCo and BigF2, BigCo and SmallCo, BigCo and SmallF1, and BigCo and Jumbo. Volcano plots exhibited that log2 ratios identified few differential proteins among large grain genotypes (Figure 2A,D). Differential proteins, revealed by our comparative analysis, included proteins associated with protein stabilization under environmental stresses, such as chaperone DnaJ 20 (DNAJ20, Q9SDN0), Dehydrin ERD10 (ERD10, P42759), Protein CutA (CUTA, P93009), and WPP domain-containing protein 2 (WPP2, Q9C500) among others, which were over-accumulated in BigCo grains compared with BigF2 and Jumbo (Figure 2A,D). The comparative analysis between BigCo and small grains (SmallCo and SmallF1) revealed additional differential proteins (Figure 2B,C), including an over-accumulation of stress-related proteins in small grains, including late embryogenesis abundant protein 31 (LEA31, Q9LJ97), late embryogenesis abundant domain-containing protein (LEA, Q9SIN3), superoxide dismutase [Cu-Zn] 1 (SODC1, P24704), 17.6 kDa class II heat shock protein (HSP176, P29830), and WPP2, compared with the large grains (Figure 2B,C). Furthermore, proteins associated with calcium signaling such as calmodulin 7 (CAM7, A0A1I9LPJ2), diacylglycerol kinase 1 (DGK1, Q39017), and calcium-binding EF-hand family protein (EFHandCa, O64866) were also over-accumulated in the small grains. In contrast, proteins associated with the acetyl-CoA biosynthetic process, including ATP-citrate synthase beta chain protein 1 (ACLB1, Q9C522) and acetyl-coenzyme A synthetase (ACS, B9DGD6), as well as hypoxia proteins such as eukaryotic aspartyl protease family protein (Aspartprot, Q9ZVS4), pyruvate decarboxylase 1 (PDC1, O82647), and PDC2 (Q9FFT4) were over-accumulated in the large grains compared to the small ones (Figure 2B,C).

**Figure 3:**
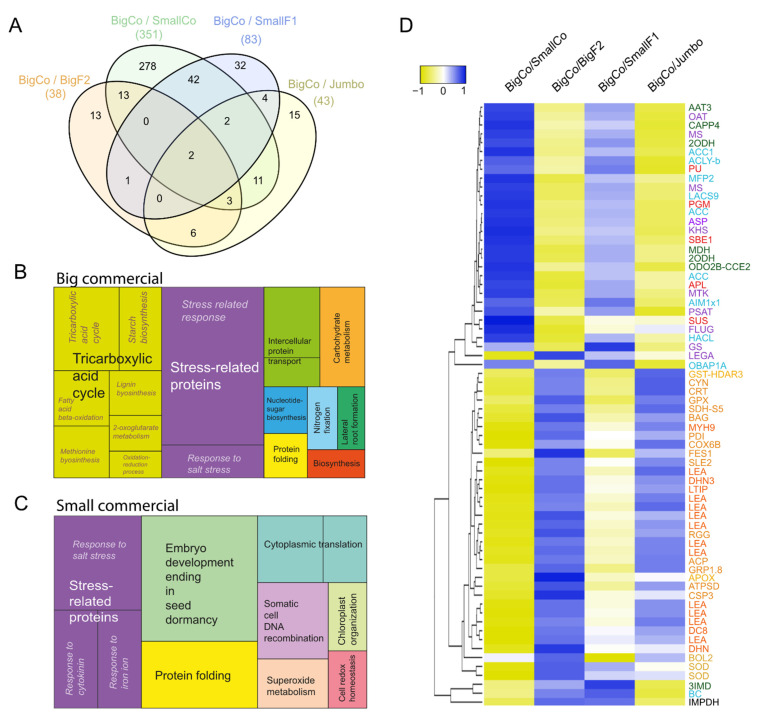
Functional annotation analysis-based GO enrichment of biological process and cluster analysis. Venn diagram of differential proteins identified in different comparisons between grain phenotypes (**A**). Treemap representation of the clustering information related to GO enrichment analysis of proteins over-accumulated in large (**B**) and small grains (**C**). Heatmap of differentially identified proteins across grain phenotypes (**D**). Abbreviation of proteins associated with starch is indicated in darker red, proteins related to lipid metabolism are in blue, TCA cycle proteins are in green, proteins related to amino acid metabolism are in purple, proteins associated with water deprivation and response to ABA are indicated in lighter red, ROS homeostasis is in aqua, and proteins related to stress, in general, are in orange.

Because of the higher number of differential proteins observed in the BigCo and SmallCo comparison, we focused on analyzing the total number of differential proteins in detail. We analyzed proteins identified in a higher proportion in BigCo compared with SmallCo grains based on GO enrichment and clustering of GO terms (Figure 3A, Table S2). This allowed us to visualize two central clusters with the term tricarboxylic acid cycle and stress-related proteins. Within this cluster, we observed proteins associated with the biosynthesis of starch, methionine, and 2-oxoglutarate metabolism connected to the production of sugar polymers and proteins. We also analyzed proteins identified in a higher proportion in SmallCo than BigCo grains (Figure 3B, Table S2). We found three main clusters, including embryo development ending in seed dormancy, stress-related proteins, and protein folding. It is worth noting that the identification of proteins associated with a response to salt stress was higher in SmallCo than BigCo grains. A pattern of associated proteins with superoxide metabolism was also evident in small grains compared to large ones (Figure 3B).

Given the differences mentioned above, we tracked the dynamics of critical proteins (Table S3). The heatmap exhibited that BigCo grains over-accumulated proteins associated with starch biosynthesis (Pullulanase 1, PULA1; Phosphoglucomutase, PGM; 1,4-α-Glucan-branching enzyme 2-2, GLGB2; Glucose-1-phosphate adenylyltransferase small subunit, GLGS; sucrose synthase 3, SUS3), lipid metabolism (Acetyl-CoA carboxylase 1, ACC1; ATP-citrate synthase β chain protein 1, ACLB; peroxisomal fatty acid β-oxidation multifunctional protein, MFP2; acetyl-coenzyme A synthetase, ACS; peroxisomal fatty acid β-oxidation multifunctional protein, AIM1; oil body-associated protein 1A, OBP1A), amino acid metabolism (ornithine aminotransferase, OAT; homoserine kinase, KHSE; 5-Methyltetrahydropteroyltriglutamate-homocysteine methyltransferase 1, METE1; Methylthioribose kinase, MTK; phosphoserine aminotransferase 1, SERB1; glutamine synthetase, GLN11), and TCA cycle (aspartate aminotransferase 3 and 5, AAT3 and AAT5, respectively; 2-oxoglutarate dehydrogenase, 2ODH; phosphoenolpyruvate carboxylase 4, CAPP4; dihydrolipoyllysine-residue succinyltransferase component of 2-oxoglutarate dehydrogenase complex 2, ODO2B; malate dehydrogenase 1, MDHM1) (Figure 3C). In contrast, small grains over-accumulated proteins associated with water deprivation and response to ABA (late embryogenesis abundant proteins, LEA6, LEA32, LEA29, LEA34; dehydrins, ERD10, DHN3; low-temperature-induced 65 kDa protein-like isoform X2, LTI65), proteins related to ROS homeostasis (glutathione S-transferase, DHAR3; protein disulfide isomerase-like 1-1, PDIL1-1; L-Ascorbate peroxidase 3, APX3; superoxide dismutase Cu-Zn 1, CSD1 and CSD12; protein BOLA2, BOLA2), and other stress-related proteins (cyanate hydratase, CYN; calreticulin, CRT; BAG family-molecular chaperone regulator, 4-like, BAG; Hsp70 nucleotide exchange factor isoform X1, Fes1A; cold shock domain-containing protein 3, CSP3; protein SLE2) (Figure 3C).

### 3.4. Untargeted Metabolomics Exhibited Additional Clues of the Nutraceutical Potential of Chickpeas

**Figure 4:**
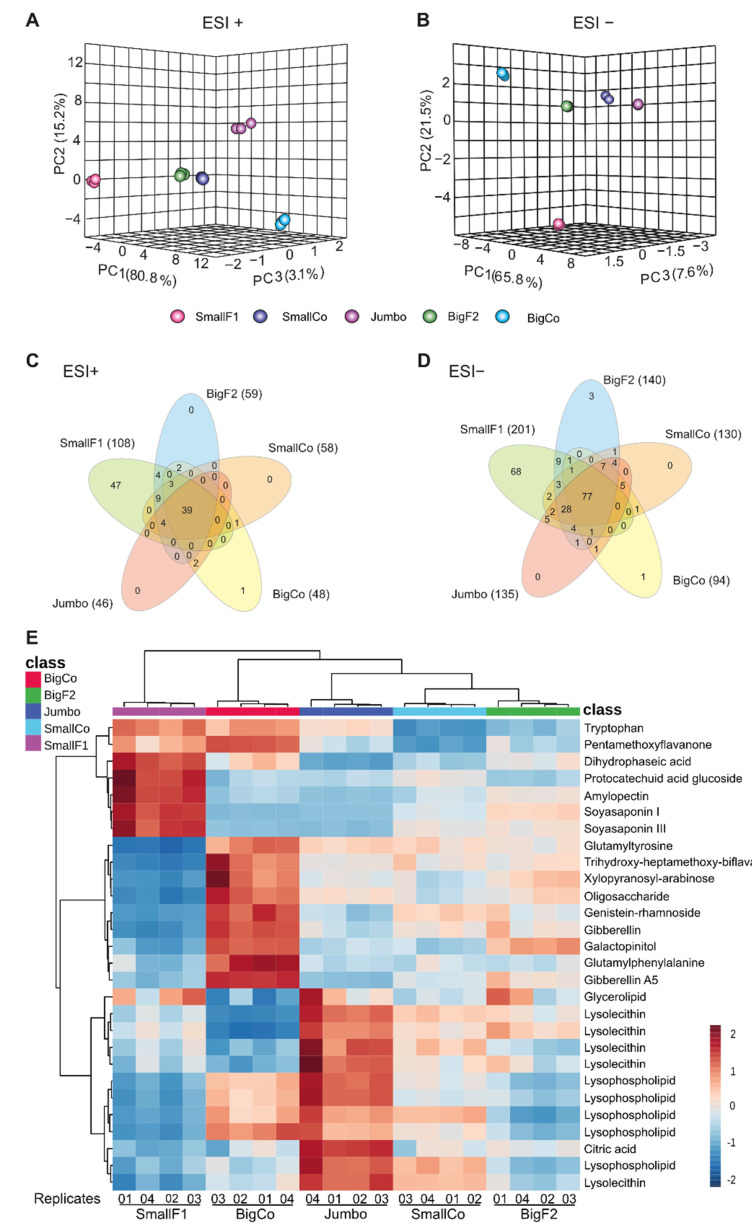
Three-dimensional principal component analyses performed with ESI positive (**A**) and negative (**B**) metabolome datasets. Venn diagrams performed with ESI positive (**C**) and negative (**D**) metabolome datasets. In (**E**), heatmap of the tentatively identified compounds of the core metabolome of studied chickpea genotypes.

To gain more information about the molecular differences among large and small chickpeas, an untargeted metabolomic approach was performed by UPLC-ESI-HRMS in positive (ESI^+^) and negative (ESI^−^) modes. Principal components analyses (PCA) of the ESI^+^ (Figure 4A) and ESI^−^ (Figure 4B) datasets exhibited contrasting groupings based on the chemical composition. In the ESI^+^ PCA, there was no clear grouping tendency; the most chemically similar genotypes were SmallCo and BigF2 (Figure 4A). In contrast, the ESI^−^ PCA clearly showed a grouping tendency among all the genotypes evaluated except for SmallF1 (Figure 4B). Venn diagrams of the spectrometric features or signals (mass/charge-retention time [*m*/*z*-Rt]) detected in the metabolic profile from each genotype showed a core metabolome of 39 and 77 signals in the ESI^+^ and ESI^−^ datasets, respectively (Figure 4C,D). The tentative identification of this core metabolome provided information on the physiological status of grains of various sizes (Figure 4E). We found carbohydrates, organic acid, nitrogen-containing compounds, phenolics, terpenoids, and lipids (Table S4). Our untargeted comparative analysis found that gibberellin and gibberellin A5 were more abundant in BigCo grains than in the other genotypes, as well as glutamyltyrosine, glutamylphenylalanine, some carbohydrates (xylopyranosyl-arabinose, galactopinitol, and an oligosaccharide) and phenolic compounds (trihydroxy-heptamethoxy-biflavan and genistein-rhamnoside) (Figure 4E). In contrast, SmallF1 genotype grains exhibited an over-accumulation of tryptophan, dihydrophaseic acid, phenolic compounds such as pentamethoxyflavanone and protocatechuic acid glucoside, the triterpenoid saponins, soy saponin I and III, and the polysaccharide amylopectin (Figure 4E). Interestingly, lipid compounds were enriched in the Jumbo genotype, exhibiting higher content of glycerolipids, lysolecithins, and lysophospholipids (Figure 4E). Citric acid was the only organic acid identified in the core metabolome, and its content was highest in the Jumbo genotype (Figure 4E).

We also noticed a high number of signals specifically detected in SmallF1 grains 47 and 68 in the ESI^+^ and ESI^−^ datasets, respectively, that were considered differential chemical markers (Figure 4C,D). These SmallF1 chemical markers included carbohydrates (mainly oligomers and polymers), nitrogen-containing compounds (mainly amino acid derivatives, an auxin, among others), phenolic compounds (mainly isoflavones), terpenoids (triterpenoid saponins), and lipids (phospholipid related and fatty acids) (Table 1).

### 3.5. Targeted Metabolomics Confirmed the Accumulation of Antioxidant Polyphenols in SmallF1

**Figure 5:**
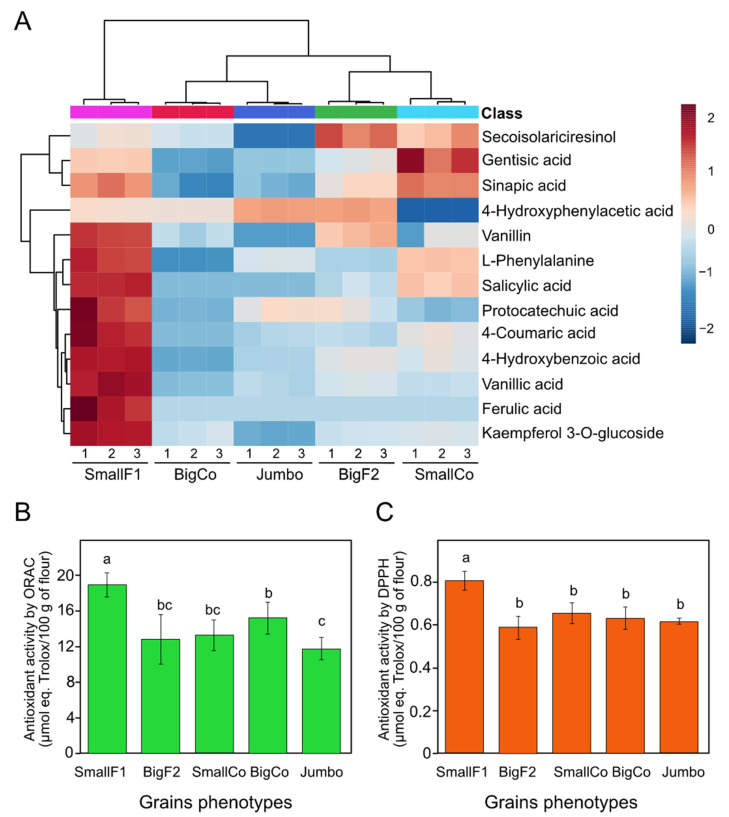
Confirmation of antioxidant capacity of small chickpea genotype. Heatmap of the 13 phenolic compounds identified and quantified in chickpea grains by targeted analysis. At the top is shown the clustering result by using the Euclidean distance and Ward algorithm in the MetaboAnalyst bioinformatic platform. Each colored cell on the map corresponds to an autoscaled concentration value (Table S5, (**A**)). Total antioxidant capacity of chickpea grain flour by oxygen radical absorbance capacity (ORAC; (**B**)) and 2-diphenyl-1-picrylhydrazyl (DPPH; (**C**)). We showed average values and the corresponding standard deviation. Different letters indicate statistical differences between samples. Data were calculated based on dry weight content.

Multiple reaction monitoring analyses based on more than 60 polyphenolics allowed us to determine the endogenous content of 13 polyphenolic-related compounds. Molecules such as vanillin, L-phenylalanine, salicylic acid, protocatechuic acid, 4-coumaric acid, 4-hydroxybenzoic acid, vanillic acid, ferulic acid, and kaempferol 3-*O*-glucoside were more abundant in SmallF1 compared to other grains (Figure 5A). SmallCo grains exhibited more gentisic and sinapic acids content than the other samples but also were over-accumulated in SmallF1. The high content of the total phenolic compounds in SmallF1 grains positively correlated with the total antioxidant capacity as determined by the DPPH and ORAC assays (Figure 5B,C), compared to other grains, which supported our proteomic and metabolomic data.

## 4. Discussion

Chickpea grains are classified into two main groups—large and small grains. The size of the grain is the most important trait for international trade because consumers demand large grains. Small grains are either sold at a low price or used as seeds for the next agricultural cycle. To our knowledge, no comparative studies have been carried out among Kabuli chickpea genotypes to determine whether there are molecular signatures or nutritionally valuable compounds related to grain size using integrative multi-omics approaches, including proteomics (isobaric tandem mass tag) and metabolomics (untargeted by accurate MS). We selected genotypes (cv. Blanco Sinaloa and Jumbo) with contrasting grain sizes, one group of small grains formed by two genotypes (SmallCo and SmallF1) and the other one formed by three large grains (BigCo, BigF2, and Jumbo). Although all five genotypes selected have the white Kabuli chickpea characteristics of white-cream color and pronounced rugosity (Figure S1A), they can be divided into two groups based on size (weight and morphometric characteristics; Figures S2 and S3).

### 4.1. Large Chickpea Genotypes Have Higher Levels of Starch, Lipid, Amino Acid, and Gibberellin Metabolism-Related Proteins

The grain filling stage is the final period during which the kernel weight is established, a trait that is directly linked to the grain yield [29]. In the BigCo grain, there was an over-accumulation of proteins associated with starch biosynthesis connected with oligosaccharide accumulation (Figure 2 and Figure 3). Similarly, previous proteomic studies of chickpea seeds and sprouts identified proteins associated with the carbon and central metabolisms such as starch synthase 1, glyceraldehyde-3-phosphate dehydrogenase, malate dehydrogenase, fructose-bisphosphate aldolase, pyruvate kinase, and ATP synthase subunit alpha [30]. The over-accumulation of proteins associated with amino acid metabolism (Figure 2 and Figure 3) overlapped with the over-accumulation of glutamyltyrosine, and glutamylphenylalanine, which are amino acid derivatives (Figure 4E). Previous proteomic and metabolomic studies in different tissues of seed wheat during the primary phase of the grain filling process exhibited the over-accumulation of isoleucine, methionine, threonine, valine, and lysine in most of the analyzed tissues, which agrees with our proteomic data in chickpea [31]. Interestingly, the presence of glutamyltyrosine and glutamylphenylalanine, plus the accumulation of oligosaccharides have been related to the highly appreciated “kokumi” taste in soybean [32]. In addition, we also found that gibberellins were over-accumulated in BigCo compared with the other genotypes (Figure 4E). Growth regulators have been previously suggested to have an essential role in modulating the grain filling process [33,34]. It was recently reported that gibberellin exogenous application of corn shank and silks improved the grain-filling rate, grain weight, and yield, as well increasing production of auxin, gibberellin, zeatin, and ABA, and activating ROS scavenger enzymes [35]. In rice, the quantitative trait locus (QTL) grain width 6 (GW6) was recently characterized, which encodes a gibberellic acid stimulated transcript (GAST) family [36]. GW6 positively controls grain size and weight, providing an increase in grain yield. Besides, auxin accumulation was connected with the regulation of starch biosynthesis during grain filling in rice, in which the knowledge regulatory network is minimal [37,38]. However, recent studies during rice development suggested that a subunit of heterotrimeric G protein (RGB) may positively regulate the expression of transcription factor OsNF-YB1, which in turn activates the OsYUC11 transcription leading to an increase in auxin level during starch accumulation [39]. We putatively identified the auxin indole-butyric acid present in the SmallF1 genotype grain (Table 1). However, the hormonal regulatory network of chickpea grain size is beyond the scope of our proteometabolomic study.

Chickpeas produce orthodox seeds, which means they will survive during ex situ conservation, where grains cope with desiccation or freezing conditions without any detrimental effect on the dormancy [40]. The mechanism associated with desiccation tolerance is turned on during late seed maturation with the accumulation of crucial proteins observed in our study. These proteins include the LEA, heat shock proteins, and ROS scavenger proteins. It is well known that there is variation among orthodox seeds in sensitivity to drying and storability that can affect grain survival [41]. In chickpeas, limited studies have been conducted to determine the association between grain size and sensitivity to desiccation and storability. However, our proteomic analysis exhibited a differential accumulation of essential proteins among grain genotypes. Small grains over-accumulated stress-related proteins related to desiccation and ABA responses (Figure 2 and Figure 3), which suggested the molecular mechanism responsible for managing drying conditions. Our proteomic data was supported by the over-accumulation of stress-related metabolites, mainly in SmallF1, related phenolics (pentamethoxyflavanone and protocatechuic acid 4-glucoside), and terpenoids (saponins soyasaponin I, and III) (Table 1). Previously, chickpea germinated in vitro, and the emulsion system boosted the antioxidative activity of extracted phenolic compounds [42]. Moreover, the protocatechuic acid 4-*O*-glucoside and 6-hydroxydaidzein were detected in the soluble free phenolic compounds [43]. Besides, in whole grain oats, it was suggested that the occurrence of the phenolic alkaloid avenanthramides, and the steroidal saponins avenacosides A and B, with potent antioxidant and anti-inflammatory effects [44].

### 4.2. Chickpea Grains Exhibited Essential Stress Response Connected to the Overproduction of Polyphenols

The significant stress response observed in SmallF1 grains led to the over-accumulation of several key secondary metabolites with nutraceutical properties (Figure 4E and Figure 5A and Table 1). Our proteomic approach also exhibited the overproduction of ROS scavenger proteins (DHAR, PDIL1-1, APX3 CSD1, CSD12, and BOLA2, Figure 2 and Figure 3). Besides, the over-accumulation of ABA-stress-related protein could suggest a tight regulation between ABA and redox balance during seed desiccation. In orthodox seeds, the acquisition of desiccation tolerance involves a complex regulatory network including several transcription factors, growth regulator signal pathways, over-accumulation of seed storage, and LEA proteins, as observed in our proteomic scrutiny (Figure 3). However, the currently limited annotation of *Cicer arietinum* genome hiders to find master molecular players associated with seed desiccation tolerance in chickpeas. Nevertheless, our findings related to the accumulation of polyphenols as a response to desiccation stress adds value to less commercialized small grains. In SmallF1, we identified 4-coumaric acid (Table 1 and Figure 5A), which has been shown to have high free radical scavenging, anti-inflammatory, antineoplastic, and antimicrobial activities [45]. In addition, several studies have suggested the antioxidant and therapeutical effects of secondary metabolites, including catechin glucoside and malvidin-acetylglucoside [46]. Isoflavones are well-known phenolic compounds, and in our study, we determined the presence of glycitein, glycitin, and malonylglycitin in SmallF1 grains (Table 1). Glycitein is an abundant isoflavone in soybean and has antioxidant, estrogenic, and anti-cancer properties [47]. Also, some triterpenoid saponins are over-accumulated in SmallF1, including soyasaponin I, III, V, and saponin D (Table 1 and Figure 4). Plant triterpenoid saponins have shown different pharmacological activities. For example, betulinic acid from *Ligustrumlucidum fructus* induced antioxidant capacity in mice liver damage induced by ethyl alcohol in vivo [48]. Besides, the ursolic acid derivative combined with kanamycin against *Escherichia coli* showed a reduction of MIC value from 128 μg/mL to 8 μg/m [49]. Moreover, oleanolic acid exhibited in vitro inhibition of the HIV-1 replication in infected human peripheral mononuclear cells (PBMC, EC_50_ value: 22.7 mM), naturally infected PBMC (EC_50_ value: 22.7 mM), and monocytes and macrophages (EC_50_ value: 57.4 mM) [50]. Soyasaponins and soyasapogenins suppressed the growth of HT-29 colon cancer cells determined by the WST-1 assay over a concentration range of 0–50 ppm [51]. Therefore, the putative identification of saponins in chickpeas could provide the starting point for finding nutraceutical molecules in chickpeas.

Corroborating the highly confident identification and higher endogenous content of essential polyphenols in smallF1 compared to other size grains underpin our proteomic and metabolomic results where stress conditions correlated with the induction in the production of polyphenols which comprise a wide range of molecules involved in the essential biological and physiological process including response to stress. Comprehensive generated information showed that the phenylpropanoid biosynthetic pathway is commonly activated under severe environmental conditions like drought, extreme temperatures, salinity, heavy metal pollution, and ultraviolet radiation [52]. We were able to identify and quantify the endogenous over-accumulation of molecules such as vanillin, l-phenylalanine, salicylic acid, protocatechuic acid, 4-coumaric acid, 4-hydroxybenzoic acid, vanillic acid, ferulic acid, and kaempferol 3-*O*-glucoside in smallF1 grains. Polyphenols are suggested to confer the ability to maintain redox homeostasis under drought and salt stress, as observed in rice and wild relatives of wheat, respectively, [53,54]. The presence of the above-mentioned compounds in the SmallF1 genotype positively correlates with the high antioxidant capacity displayed by this genotype (Figure 5). Previous studies showed that colored chickpea grains have significant antioxidant values than cream and beige seed [15], but in our study, all genotypes analyzed have the same cream color (Figure S1A). Therefore, we could suggest that SmallF1 grains can over-produce polyphenols probably due to the response to stress conditions (Figure 5A). The profiling of these polyphenols during germination as it was previously reported for formononetin and biochanin [16], as well as in other experimental conditions, could contribute to unraveling the regulation of the biosynthesis of these bioactive molecules.

### 4.3. Alternative Application of Small Chickpeas as Nutraceutical Source

The dynamic of grain commercialization is focused on yield percentage. Therefore, bigger grains provide a better economic profit, which is justified by the need for protein, fiber, sugar, and lipids. However, after two years of the pandemic due to the SARS-CoV-2 virus and its variants, emerging diseases, including severe acute hepatitis in children and monkeypox, make us reconsider the continuous search for healthy and functional foods. Animal, human, and epidemiologic studies strongly suggest that polyphenols have antioxidant and anti-inflammatory properties that probably prevent and exert a therapeutic effect in several illnesses, for example, in cardiovascular disease, neurodegenerative disorders, cancer, and obesity [55]. However, in whole grains, bioactive molecules have long been undervalued [56]. In buckwheat (*Fagopyrum esculentum* Moench), amaranth (*Amaranthus* L.), and quinoa (*Chenopodium quinoa* Willd.), spectrophotometric approaches and free radical scavenging activity 2,2-diphenyl-1-picrylhydrazyl (DPPH) determinations suggest that the cultivars Bamby (buckwheat), Annapurna (amaranth), and Quinua (quinoa) are excellent sources of bioactive compounds [57]. Cereals contain free and bound forms of polyphenols, and some examples include ferulic, protocatechuic, gallic, vanillic, and syringic acids, among others [56]. In chickpeas, we confidently identified and quantified the endogenous content of vanillin, l-phenylalanine, salicylic, protocatechuic acid, 4-coumaric, 4-hydroxybenzoic, vanillic, ferulic acids, and kaempferol 3-*O*-glucoside.

Phenolic compounds are widely distributed in plants and exhibit multiple bioactivities including antitumoral, antioxidant, and anti-inflammatory, just to mention some examples [58,59,60]. The three most abundant phenolic compounds identified and quantified in the Kabuli chickpea grains evaluated were 4-hydroxyphenylacetic acid, 4-hydroxybenzoic acid, and gentisic acid. The phenolic compound 4-hydroxyphenylacetic acid was determined mainly in big grains (Table S5 and Figure 5A), and it has exhibited anti-inflammatory and hepatoprot in induced lung injury in rats [61] and in acetaminophen-induced liver injury in mice [62], respectively. On the other hand, 4-hydroxybenzoic acid and gentisic acid were determined over-accumulated in small grains (Table S5 and Figure 5A). 4-Hydroxybenzoic acid exhibited a hypoglycemic effect in rats [63] and an antimicrobial effect [64], while gentisic acid has demonstrated a beneficial effect on human health as an analgesic, anti-inflammatory, antigenotoxic, cardioprotective, hepatoprotective, neuroprotective, nephroprotective, antimicrobial and antioxidant agent [65]. Therefore, the occurrence of the above-mentioned molecules highlights the healthy value as functional food of small grains and other locally produced grains, in which the production of polyphenols surpasses the average content determined in big chickpeas, being a good option for nutraceuticals supplementation, especially for people with vegetable source-based diets or low-income sector in developing countries [66].

## 5. Conclusions

The global market demands big grain sizes, and the Blanco Sinaloa and Jumbo Mexican chickpea varieties meet this international standard. As chickpea plants are self-pollinated, agriculture can generate their seeds for the next agriculture cycle providing savings and additional gains. Due to its self-pollination, chickpea segregates genetic and phenotypic features in every agricultural cycle. Thus, farmers always collect two-grain sizes, including small and big ones. The big grain goes to the international market and the small grains for local sale. Thus, there is great interest in finding molecules with nutraceutical properties in small grains, which could provide additional values to this type of phenotype. Using classical and cutting-edge technologies, we showed that protein content was not correlated with the morphometric characteristic related to grain sizes, but protein profiles were contrasting between small and big grains. Proteomics and metabolomics showed that in big grains, starch biosynthesis and gibberellins were more abundant than in small grains, but in the latter, stress signaling correlated with phenolic compounds content and antioxidant activity. Therefore, it represents a healthy source of nutraceuticals. Also, due to global warming, high cost of production, and pathogens, the chickpea breeders are developing new varieties or cultivars focused on increasing grain yield, pathogen resistance, and environmental factors such as drought. Our study showed that some germplasms have better nutraceutical properties and could be used as genetic resources in molecular chickpea breeding programs to develop biofortified grains.

## Figures and Tables

**Table 1:** Putative identification of compounds only detected in SmallF1 chickpea grains.

RT	*m*/*z*	Compound	Adduct	Adduct *m*/*z* *	Mass Error	Chemical Group
**Carbohydrates**						
0.44	245.0432	Sedoheptulose	[M + Cl]^−^	245.0434	−0.8	Ketoheptose
0.47	705.1849	Stachyose	[M + K]^+^	705.1855	−0.9	Tetrasaccharide
0.50	527.1593	Dextrin	[M + Na]^+^	527.1583	1.9	Polysaccharide
1.45	451.1423	Xylopyranosyl-rhamnopyranosyl-arabinose	[M + Na]^+^	451.1422	0.2	Oligosaccharide
1.82	383.1566	Tri-methyl-mannobiose	[M − H]^−^	383.1559	1.8	Disaccharide derivative
2.71	419.1524	Methylbutanoyl-apiosylglucose	[M + Na]^+^	419.1524	0.0	O-acyl carbohydrate
3.77	435.2243	Ethyl cellulose	[M − H_2_O − H]^−^	435.2230	3.0	Polysaccharide
**Nitrogen-containing Compounds**						
0.42	104.1078	Choline	[M + H]^+^	104.1075	2.9	Phospholipid precursor
1.14	134.0472	Adenine	[M − H]^−^	134.0467	3.7	Aminopurine
2.65	247.1441	Feruloylputrescine	[M − H_2_O + H]^+^	247.1447	−2.4	Polyamine derivative
2.79	186.0917	Indole-butyric acid	[M − H_2_O + H]^+^	186.0919	−1.1	Auxin
3.36	250.0726	Feruloylglycine	[M − H]^−^	250.0721	2.0	Amino acid derivative
7.03	268.0369	Indoleglycerol phosphate	[M − H_2_O − H]^-^	268.0375	−2.2	Amino acid derivative
**Phenolics**						
0.45	451.1239	Catechin glucoside	[M − H]^−^	451.1246	−1.6	Flavanol glucoside
0.54	601.1380	Peonidin	[2M − H]^−^	601.1362	3.0	O-Methylated anthocyanidin
1.46	331.0681	Galloyl glucose	[M − H]^−^	331.0671	3.0	Gallic acid glucoside
1.71	165.0551	Coumaric acid	[M + H]^+^	165.0546	3.0	Hydroxycinnamic acid
7.04	447.1292	Glycitin	[M + H]^+^	447.1286	1.3	Glycosyloxyisoflavone
7.70	555.1107	Malonylglycitin	[M + Na]^+^	555.1109	−0.4	Glycosyloxyisoflavone
8.65	533.1292	Malvidin-acetylglucoside	[M − H]^−^	533.1306	−2.6	Anthocyanin derivative
9.69	285.076	Glycitein	[M + H]^+^	285.0757	1.1	Methoxyisoflavone
**Terpenoids**						
7.95	957.505	Soyasaponin V	[M − H]^−^	957.5065	−1.6	Triterpenoid saponin
10.26	925.5156	Saponin D	[M − H]^−^	925.5166	−1.1	Triterpenoid saponin
**Lipids**						
11.53	316.2849	Dehydrophytosphingosine	[M + H]^+^	316.2846	0.9	Sphingolipid
12.38	315.0478	Glycerophosphoinositol	[M − H_2_O − H]^−^	315.0481	−1.0	Phospholipid derivative
13.40	295.2272	Epoxy-hydroxystearate	[M − H_2_O − H]^−^	295.2273	−0.3	Epoxy fatty acid
13.98	255.2325	Hexadecanoic acid	[M − H]^−^	255.2324	0.4	Fatty acid

RT: Retention time in minutes; *m*/*z* in Daltons; * calculated value; mass error in ppm.

## Data Availability

Data is contained within the article and Supplementary Material. Raw proteomic data are available via ProteomeXchange with identifier PXD030279. Raw metabolic data are available under request.

## Supplementary Materials

The following supporting information can be downloaded at: https://www.mdpi.com/article/10.3390/antiox11101850/s1. Figure S1: Chickpea seed (*Cicer arietinum* L.) phenotypes, showing ventral (left) and lateral (right) characteristics. SmallF1 = small farmer 1; BigF2 = big farmer 2; SmallCo = small commercial; BigCo = large commercial (A), theorical volume of grains = length * thickness * width (B), and measurements of the length (C), width (D), thickness (E), and weight (F) of seeds. The data show the mean ± standard deviation. Different letters above the bars indicate significant differences. Figure S2: Matrix of correlation coefficients. Protein content = pro, weight, length, width, thickness, and volume correspond to a morphometric characteristic of chickpea grains. Numbers inside the boxes are the coefficient of correlation, and the font size is proportional to the strength of the correlation. Level of significance is indicated by asterisks (*p*-values < 0.001 = 3*, 0.001 = 2*, 0.01 = 1*, 0.05 = 0* ) (A), correlation coefficient matrix of chickpea proximate variables. Moisture = Most, Protein content = Pro, Lipids = Lip, Crude fiber = Fiber, and nitrogen free extract = NFE. Numbers inside the boxes indicate the coefficient of correlation, and the font size is proportional to the strength of the correlation. Level of significance is indicated by asterisks (*p*-values < 0.001 = 3*, 0.001 = 2*, 0.01 = 1*, 0.05 = 0* (B). Table S1: Proximate composition of chickpea grains. Detail information of untargeted metabolomic data in chickpea grains. Table S2: Functional annotation analysis-based GO enrichment of biological proceeds and cluster analysis of proteins over-accumulated in big (A) and small grains (B). Table S3: Key differential proteins identified in proteomic comparative analysis-based TMT-SPS-MS^3^. Data associated with figure 3D. Table S4: Putative identification of core compounds detected in chickpea grains. Table S5: Endogenous content of phenolic compounds in chickpea grains. Table S6: Conditions for each compound used in MRM (Multiple Reaction Monitoring).

## Appendix A. Detail Proteomics and Metabolomics Methodologies

### Appendix A.1. Proteomics Analyses

#### Appendix A.1.1. Sample Preparation

Our general protocol was based on Monribot et al. (2019) using the phenol-based extraction method with few modifications. For this, we pooled 2 g of chickpea powder, previously processed with a food processor (Nutribullet). It was suspended in three volumes of ice-cold extraction buffer (150 mM Tris-HCl, pH 8.0, 100 mM KCl, 0.7 M sucrose, 0.3% Triton X-100, 1% DTT, 1 g/10 g fresh weight of PVPP and 20 L/g fresh weight of protease inhibitor cocktail; Sigma-Aldrich, St. Louis, MO, USA). The samples were then homogenized with a tissue homogenizer (TissueTearor™, BioSpec Products, Inc., Bartlesville, OK, USA). The resulting solution was combined with an equal volume of phenol solution (equilibrated with 10 mM Tris-HCl, pH 8.0, and 1 mM EDTA, Sigma, St. Louis, MO, USA) and shaken on ice for 30 min at 4 °C. The mixture was then centrifuged at 10,000× g for 30 min at 4 °C, the upper phenolic phase collected, and the proteins precipitated with four volumes of ice-cold acetone overnight at −20 °C. The resulting pellet was dried at room temperature for 15 min and dissolved with 200 µL of 50 mM phosphate-buffered saline (PBS, pH 7.4) containing 1% of SDS. Protein concentration was determined using the BCA™ Assay Kit (Pierce; Rockford, IL, USA), using bovine serum albumin as a standard.

Following reduction and alkylation, 100 µg of protein were digested with 100 mM Tris (2-carboxyethyl) phosphine (TCEP) stock solution for a final concentration of 10 mM for 45 min at 60 °C. Then 300 mM of iodoacetamide (IA) stock solution was added for a final concentration of 30 mM and incubated for one hour at room temperature in darkness. We then quenched the remaining IA with 300 mM DTT stock solution for a final concentration of 30 mM for 10 min. Following overnight protein precipitation with acetone, the protein pellet was resuspended with 100 µL of 50 mM triethylammonium bicarbonate (TEAM) containing 0.1% SDS. The proteins were then digested with trypsin (Trypsin Gold, Mass Spectrometry Grade, Promega, Madison, WI, USA) and labeled with TMT 10plex reagent according to the manufacturer’s instructions (AB-Sciex; Foster City, CA, USA). For SmallF1, we used 126 and 127N; for BigF2, we used 127C and 128N; for SmallCo, we used 128C and 129N; for BigCo, we used 129C and 130N; and for Jumbo, we used 130C and 131. Labeled samples were pooled and fractionated using strong cation exchange and high pH reversed-phase C18 cartridges (Thermo Scientific). Each fraction was desalted with C18 cartridges and dried using a CentriVap (Labconco, Kansas, MI, USA).

#### Appendix A.1.2. Nano-LC-MS/MS, Synchronous Precursor Selection (SPS)-MS3 and Data Analysis

Dried samples were redissolved with 0.1% formic acid in LC-MS grade water (solvent A), and 5 µL of this solution was injected into a nano-LC platform (UltiMate 3000 RSLC system, Dionex, Sunnyvale, CA, USA) through a nanoviper C18 trap column (3 µm, 75 µm × 2 cm, Dionex), and separated on an EASY spray C-18 RSLC column (2 µm, 75 µm × 25 cm) at a flow rate of 300 nL/min. The mobile phase gradient was as follows: a 10 min gradient was set using Solvent A and 0.1% formic acid in 90% acetonitrile (Solvent B), 10 min of Solvent A, 7–20% Solvent B over 25 min, 20% Solvent B for 15 min, 20–25% Solvent B over 15 min, 25–95% Solvent B over 20 min, and 8 min of Solvent A. The nano-LC was interfaced with an Orbitrap Fusion Tribid (Thermo-Fisher Scientific, San Jose, CA, USA) mass spectrometer equipped with an “EASY Spray” nano ion source (ThermoFisher Scientific, San Jose, CA, USA). The mass spectrometer was operated in a positive ionization mode with the nanospray voltage set at 3.5 kV and a source temperature of 280 °C. For external calibration, we used caffeine, Met-Arg-Phe-Ala (MRFA) and Ultramark 1621. Full MS scans were carried out in the Orbitrap analyzer at resolution 120,000 (FWHM), scan range 350–1500 *m*/*z*, AGC of 2.0 × 10^5^, maximum injection time of 50 ms, intensity threshold of 5.0e3, dynamic exclusion 1 at 70 s and mass tolerance of 10 ppm. For MS2 analysis, the 20 most abundant MS1 were isolated with charge states set to 27. Fragmentation parameters included CID with 35% collision energy and activation Q of 0.25, AGC of 1.0 × 10^4^, maximum injection time of 50 ms, precursor selection mass range of 400–1200 *m*/*z*, precursor ion exclusion width low 18 *m*/*z* and high 5 *m*/*z*, isobaric tag loss TMT and detection was carried out in the ion trap and the MS3 spectra were acquired using an SPS of 10 isolation notches. MS3 precursors were fragmented by HCD with 65% of collision energy and analyzed using the Orbitrap at a resolution of 60,000 with a scan range of 120–500 *m*/*z*, isolation window of 2 *m*/*z*, 1.0 × 10^5^ AGC, and a maximum injection time of 120 ms with 1 microscan.

The spectra acquired by MS/MS and (SPS)-MS 3 were analyzed with the program Proteome Discoverer 2.1 (Thermo Fisher Scientific Inc.). Data were processed using the search engines Amanda, Mascot server (version 2.4.1, Matrix Science, Boston, MA, USA), and Sequest HT algorithm, in which searches were directed against chickpea UniProt Reference proteome (UP000087171). As search parameters, we considered full-tryptic protease specificity, two missed cleavages, carbamidomethylation of cysteine (+57.021 Da) and TMT 9-plex N-terminal/lysine residues (+229.163 Da). Moreover, we considered methionine oxidation (+15.995 Da) and deamidation in asparagine/glutamine (+0.984 Da) as dynamic modifications. Protein identification was carried out at a lower resolution in the linear ion trap with tolerances of ±10 ppm and ±0.6 Da. Peptide hits were filtered for a maximum of 1% FDR using the Percolator algorithm (Käll, Canterbury, Weston, Noble & MacCoss, 2007). In addition, TMT quantification was performed at the MS3 level in the Orbitrap analyzer with the precursor co-isolation filter set to 45%. Data are available via ProteomeXchange with identifier PXD030279 (https://www.ebi.ac.uk/pride/ (Last accessed on 1 July 2022)).

#### Appendix A.1.3. Bioinformatic Analysis

Proteins and peptides abundance was generated on the Proteome Discover 2.4 platform. Normalization was carried out based on the total peptide amount. After that , we determined the fold change of protein abundance in the ratio BigCo/BigF2, BigCo/SmallCo, BigCo/SmallF1, and BigCo/Jumbo. Differential proteins [fold change > 1.5 and <0.66 (log2 fold change > 0.59 and <−0.59)] were analyzed based on biological process gene ontology (GO) enrichment using the platform DAVID Bioinformatics Resources 6.8 (https://david.ncifcrf.gov/summary.jsp (Last accessed on 1 July 2022)), and the resulting GO terms were then summarized by finding a representative subset with a simple clustering algorithm that relies on semantic similarity measures using REVIGO software (http://revigo.irb.hr/ (Last accessed on 1 July 2022)). We used chickpea Uniport annotation as well as protein homologs to Arabidopsis. The output data were presented using Treemaps, displaying a two-level hierarchy of GO terms—each rectangle is a single cluster representative, and the representatives are joined into ‘superclusters’ of loosely related terms.

### Appendix A.2. Metabolomics Analysis

#### Appendix A.2.1. Untargeted Metabolomics Analyses

The untargeted metabolomics analyses were performed in an ultra-high-resolution chromatograph (UPLC) (Waters™, Class I) coupled with a high-resolution QTOF mass spectrometer (HRMS) (Waters™, Synapt G2Si), as previously reported (MonribotVillanueva et al., 2019). An Acquity BEH column (Waters™) (1.7 µm, 2.1 × 50 mm) was used for the chromatographic separation, with a column oven temperature of 40 °C and sample temperature of 15 °C. The mobile phase was a combination of water (A) and acetonitrile (B), both with 0.1% of formic acid (all solvents used were MS grade from SIGMA). The gradient used began with 1% of B, followed by a linear gradient from 1 to 80% of B over 13 min, then isocratic for 1 min at 80% of B, and, finally, a linear gradient from 80 to 1% of B over 1 min. The total run time was 20 min. The flow rate was 0.3 mL/min, and 2 µL of sample solution were injected. For the spectrometric analyses, electrospray ionization (ESI) was used in positive and negative modes. The capillary, sampling cone, and source offset voltages were 3000, 40 and 80 V, respectively. The source and desolvation temperatures were 100 and 20 °C, respectively. The desolvation gas flow rate was 600 L/h and the nebulizer pressure was 6.5 Bar (650,000 Pa). Leucine-enkephaline was used as a lock mass. The mass spectrometer was set with a mass range of 50–1200 Da using an MS e method. The collision energy of Function 1 was 6 V, while for Function 2, we used a ramp from 10 to 30 V. The scan time was 0.5 s. Metabolomics data were first processed with MassLynx (Waters™) and MarkerLynx (Waters™), then the bioinformatic analyses were performed on the MetaboAnalyst bioinformatic platform (https://www.metaboanalyst.ca/MetaboAnalyst/home.xhtml (Last accessed on 1 July 2022)).

#### Appendix A.2.2. Targeted Metabolomics Analyses (Phenolic Compounds)

##### Sample Preparation

Samples were dissolved in methanol (50 mg/mL), filtered with 0.5 μm PTFE membranes, and placed in 2 mL UPLC vials.

##### Multiple Reaction Monitoring (MRM)

Phenolic compounds were identified and quantified in the same fruit samples with a dynamic multiple-reaction monitoring method using a UPLC (Agilent Technologies, 1290) coupled to a QqQ mass spectrometer (Agilent Technologies, 6460) and based on protocols previously described by our research group (Juárez-Trujillo et al., 2018; Monribot-Villanueva et al., 2019). For the chromatographic separation, a Zorbax SB-C18 column (Agilent Technologies) (1.8 µm, 2.1 × 50 mm) with a column oven temperature of 40 °C was used. The mobile phases were water (A) and acetonitrile (B), both with 0.1% of formic acid (all solvents were MS grade from SIGMA). The gradient used begins with 1% of B, then a linear gradient from 1 to 40% of B over 40 min and a linear gradient from 40 to 90% of B over 2 min, then isocratic for 2 min at 90% of B and, finally, a linear gradient from 90 to 1% of B over 2 min. The total run time was 47 min. The flow rate was 0.1 mL/min, and 1 µL were injected. For the spectrometric analyses, the ESI source was used in positive and negative modes. The desolvation temperature was 300 °C, the cone gas (N 2) flow rate was 5 L/min, and the nebulizer pressure was 45 psi (310,264 Pa). The sheath gas temperature and flow rate values were 250 °C and 11 L/min, respectively. The capillary voltage was 3500 V, and the nozzle voltage was 500 V. All data were processed using the MassHunter software (Agilent Technologies). For quantification, calibration curves were constructed from 0.25 to 17 µM for each compound. The r 2 values for quadratic regressions were 0.99. Data were processed with the MassHunter software (Agilent Technologies). The heatmap shows the clustering result by using the Euclidean distance and Ward algorithm in the MetaboAnalyst bioinformatic platform.

The protocol used was a dynamic MRM (Multiple Reaction Monitoring). The conditions for each compound are described in Table S6.
