# Supplementary material for: Proteometabolomic Analysis Reveals Molecular Features Associated with Grain Size and Antioxidant Properties amongst Chickpea (Cicer arietinum L.) Seeds Genotypes
Source: Antioxidants (Basel). 2022 Sep 20;11(10):1850. doi: 10.3390/antiox11101850 (PMC9598084; doi:10.3390/antiox11101850)
Supplement: Supplementary file 1 [file antioxidants-11-01850-s001.zip › Table S1.pdf]

Table S1. Proximate composition of Chickpea grains. Number in bold and underlined are highest and lowest data. Different letters or asterisks refer to significant differences.

Genotype	Protein (%)		Moisture (%)		Lipids (%)		Crude fiber (%)		Ash (%)		Nitrogen free extract (%)	
	Mean	SD	Mean	SD	Mean	SD	Mean	SD	Mean	SD	Mean	SD
SmallF1	19.57	0.14^{b}	8.71	0.14*	11.01	0.53*	4.06	0.62^{c}	3.02	0.09	53.62	0.86^{c}
BigF2	21.49	0.52^{a}	7.92	0.16	5.50	0.16	6.24	0.41^{b}	3.11	0.09	55.73	0.88^{b}
SmallCo	18.50	0.26^{b}	7.70	0.13	5.40	0.36*	6.26	0.21^{b}	3.23	0.16	58.84	0.40^{a}
BigCo	16.96	0.85^{c}	7.82	0.15	6.72	0.07	5.65	0.10^{b}	3.18	0.14	59.66	0.88^{a}
Jumbo	19.62	0.22^{b}	7.61	0.11	6.64	0.51	7.33	0.23^{a}	3.19	0.05	55.60	0.17^{b}
