# Supplementary material for: Proteometabolomic Analysis Reveals Molecular Features Associated with Grain Size and Antioxidant Properties amongst Chickpea (Cicer arietinum L.) Seeds Genotypes
Source: Antioxidants (Basel). 2022 Sep 20;11(10):1850. doi: 10.3390/antiox11101850 (PMC9598084; doi:10.3390/antiox11101850)
Supplement: Supplementary file 1 [file antioxidants-11-01850-s001.zip › Table S2.pdf]

Table S2. Functional annotation analysis-based GO enrichment of biological proceeds and cluster analysis of proteins over-accumulated in big (A) and small grains (B).

**Big grains (A)**						
% WARNING - This is exported REVIGO data useful only for the specific purpose of constructing a TreeMap visualization.						
% Do not use this table as a general list of non-redundant GO categories	as it sets an extremely permissive					
% threshold to detect redundancies (c=0.10) and fill the 'representative' column	while normally c>=0.4 is recommended.					
% To export a reduced-redundancy set of GO terms	go to the Scatterplot & Table tab	and export to CSV from there.				
term_ID	description	frequencyInDb	log10 pvalue	uniqueness	dispensability	representative
GO:0006099	tricarboxylic acid cycle	0.47%	-4.1085	0.619	0	tricarboxylic acid cycle
GO:0009086	methionine biosynthetic process	0.33%	-2.1718	0.534	0.4	tricarboxylic acid cycle
GO:0006103	2-oxoglutarate metabolic process	0.02%	-1.3878	0.66	0.319	tricarboxylic acid cycle
GO:0019252	starch biosynthetic process	0.01%	-2.6288	0.708	0.125	tricarboxylic acid cycle
GO:0009809	lignin biosynthetic process	0.00%	-1.7186	0.774	0.194	tricarboxylic acid cycle
GO:0055114	oxidation-reduction process	15.06%	-1.0575	0.77	0.216	tricarboxylic acid cycle
GO:0006635	fatty acid beta-oxidation	0.08%	-2.3202	0.597	0.354	tricarboxylic acid cycle
GO:0006886	intracellular protein transport	1.20%	-2.9283	0.92	0	intracellular protein transport
GO:0016192	vesicle-mediated transport	1.09%	-1.2461	0.92	0.32	intracellular protein transport
GO:0046686	response to cadmium ion	0.03%	-12.0205	0.883	0	response to cadmium ion
GO:0009651	response to salt stress	0.04%	-2.609	0.839	0.231	response to cadmium ion
GO:0009058	biosynthetic process	31.61%	-1.2613	0.93	0.021	biosynthesis
GO:0006457	protein folding	0.90%	-1.4729	0.91	0.032	protein folding
GO:0009226	nucleotide-sugar biosynthetic process	0.14%	-1.483	0.824	0.05	nucleotide-sugar biosynthesis
GO:0010311	lateral root formation	0.00%	-1.2676	0.891	0.051	lateral root formation
GO:0005975	carbohydrate metabolic process	5.26%	-3.3645	0.868	0.069	carbohydrate metabolism
GO:0009399	nitrogen fixation	0.07%	-1.3878	0.885	0.084	nitrogen fixation

**Small grains (B)**						
% WARNING - This is exported REVIGO data useful only for the specific purpose of constructing a TreeMap visualization.						
% Do not use this table as a general list of non-redundant GO categories	as it sets an extremely permissive					
% threshold to detect redundancies (c=0.10) and fill the 'representative' column	while normally c>=0.4 is recommended.					
% To export a reduced-redundancy set of GO terms	go to the Scatterplot & Table tab	and export to CSV from there.				
term_ID	description	frequencyInDb	log10 pvalue	uniqueness	dispensability	representative
GO:0006457	protein folding	0.90%	-4.2848	0.9	0	protein folding
GO:0009651	response to salt stress	0.04%	-4.5498	0.53	0	response to salt stress
GO:0010039	response to iron ion	0.01%	-2.3055	0.565	0.371	response to salt stress
GO:0009735	response to cytokinin	0.01%	-2.3396	0.604	0.22	response to salt stress
GO:0009793	embryo development ending in seed dormancy	0.02%	-7.9431	0.85	0	embryo development ending in seed dormancy
GO:0016444	somatic cell DNA recombination	0.01%	-2.9189	0.855	0.024	somatic cell DNA recombination
GO:0009658	chloroplast organization	0.02%	-1.4237	0.852	0.025	chloroplast organization
GO:0006801	superoxide metabolic process	0.14%	-1.93	0.84	0.038	superoxide metabolism
GO:0045454	cell redox homeostasis	0.86%	-1.1955	0.867	0.062	cell redox homeostasis
GO:0002181	cytoplasmic translation	0.06%	-2.4479	0.871	0.087	cytoplasmic translation
GO:0006123	mitochondrial electron transport, cytochrome c to oxygen	0.01%	-1.6315	0.81	0.166	cytoplasmic translation
