# Supplementary material for: Proteometabolomic Analysis Reveals Molecular Features Associated with Grain Size and Antioxidant Properties amongst Chickpea (Cicer arietinum L.) Seeds Genotypes
Source: Antioxidants (Basel). 2022 Sep 20;11(10):1850. doi: 10.3390/antiox11101850 (PMC9598084; doi:10.3390/antiox11101850)
Supplement: Supplementary file 1 [file antioxidants-11-01850-s001.zip › Table S3.pdf]

Table S3. Key differential proteins identified in proteomic comparative analysis-based TMT- SPS-MS^3**.** Data associated with figure 3D**.**

Checkpea	Arabidopsis homologs	Protein names	Log2 BigCo/SmallCo	Log2 BigCo/BigF2	Log2 BigCo/SmallF1	Log2 BigCo/Jumbo
A0A1S2XH76	Q9SGH6	Alpha-dioxygenase 1 (Alpha DOX1) (EC 1.14.99.-) (	0.50496595	-0.1965366	0.27386754	-0.0241967
A0A1S2Y5V8	Q9C826	Xanthoxin dehydrogenase (EC 1.1.1.288)	0.05212273	-0.3013044	-0.1501403	0.01402956
A0A1S2YI47	Q39026	Mitogen-activated protein kinase 6 (AtMPK6) (MAP kinase 6) (EC 2.7.11.24)	0.5322928	0.17250951	0.39715673	-0.1068914
A5X7B9	Q39023	Mitogen-activated protein kinase 3 (AtMPK3) (MAP kinase 3) (EC 2.7.11.24)	0.48347024	-0.0010873	0.32701313	0.04754745
A0A1S3E9R6	F4JZY1	COP1-interactive protein 1	-0.2991496	0.01452611	0.10489519	0.04267062
A0A1S2Y229	Q940H6	Serine/threonine-protein kinase SRK2E (EC 2.7.11.1)	0.41119543	-0.1392512	-0.0127673	-0.1669296
Q8L5Q6	O22850	Probable glutathione peroxidase 3, mitochondrial (EC 1.11.1.9)	0.12382392	0.1698014	-0.2746883	-0.0647751
A0A1S2YQ46	Q93VR4	MLP-like protein 423	-0.1120235	0.42553818	-0.1892971	-0.0188879
A0A1S2XKS3	Q9ZQ80	Nodulin-related protein 1 (AtNRP1) (RPS2-interacting protein 11)	-1.0187994	0.2403321	-0.7680418	0.1560192
A0A1S2XED 8	Q38882	Phospholipase D alpha 1 (AtPLDalpha1) (PLD alpha 1) (EC 3.1.4.4)	0.48583482	0.01720427	0.21870476	0.06213339
A0A1S2XQL 9	Q9C5Z2	Eukaryotic translation initiation factor 3 subunit H (eIF3h) (Eukaryotic translation initiation factor 3 subunit 3) (eIF-3-gamma) (eIF3 p38 subunit)	0.47845533	-0.1762574	0.30323489	-0.1254189
A0A1S2XUA 5	Q8L4B2	Nuclear transcription factor Y subunit C-9 (AtNF-YC-9) (Transcriptional activator HAP5C)	0.1153198	-0.1753026	-0.4128318	0.0158296
A0A1S2YGY 7	Q42546	SAL1 phosphatase (3'(2'),5'-bisphosphate nucleotidase 1) (EC 3.1.3.7) (3'(2'),5'-bisphosphonucleoside 3'(2')-phosphohydrolase 1)	-0.0955443	0.02153316	-0.1514831	0.03774836
A0A1S2YIG9	Q9SSL1	Protein C2-DOMAIN ABA-RELATED 7	0.16794464	0.0052943	-0.3646237	-0.0313642
A0A1S2YDP0	Q9XFK7	Protein MOTHER of FT and TFL1	0.38596651	-0.192271	-0.0327588	-0.083736
A0A1S3DYR 5	F4IVM7	Non-specific serine/threonine protein kinase (EC 2.7.11.1)	0.55834421	-0.0445006	0.04955042	0.2350857
A0A1S2YLS1	Q02973	Em-like protein GEA6	-1.5704823	0.12670394	-0.492603	0.09983954
A0A1S2YHK 8	Q9FNK4	Ornithine aminotransferase, mitochondrial (EC 2.6.1.13)	0.64582208	-0.0731045	0.36430292	-0.2967347

A0A1S2Z041	Q9M060	Eukaryotic translation initiation factor 6-2 (AteIF-6;2)	-0.2030919	-0.2824743	-0.4782193	-0.4758088
A0A1S2Z701	Q9SYT0	Annexin D1 (AnnAt1) (Annexin A1)	0.43691362	-0.1622683	0.18683525	-0.0733399
A0A1S2XE65	Q9LVC5	Glucose-6-phosphate 1-epimerase (EC 5.1.3.15)	0.7249457	0.08033371	0.36447715	-0.2106376
A0A1S2Z6F7	Q9FND9	Probable galactinol--sucrose galactosyltransferase 5 (EC 2.4.1.82)	-0.2347321	-0.0955263	-0.1416227	-0.2072514
A0A1S2YBQ7	Q70DU8	Aldehyde dehydrogenase family 3 member H1 (AtALDH4) (Ath-ALDH4) (EC 1.2.1.3)	0.64664244	0.01316267	0.60897304	0.01430742
A0A1S2XGM0	Q9SYG7	Aldehyde dehydrogenase family 7 member B4 (EC 1.2.1.3) (Antiquitin-1)	0.50930374	-0.2939561	0.21884598	0.00806681
A0A1S2XPP7	O04499	2,3-bisphosphoglycerate-independent phosphoglycerate mutase 1 (BPG-independent PGAM 1) (Phosphoglyceromutase 1) (EC 5.4.2.12) (PGAM-I 1)	0.39766737	-0.0464101	0.18187894	-0.0326432
A0A067XTI7	Q9SYK9	UDP-glycosyltransferase 74E2 (EC 2.4.1.-)	0.65057656	-0.0077209	0.4181744	-0.0441092
A0A1S2XXH2	Q9LJ97	Late embryogenesis abundant protein 31 (LEA 31) (Protein RESPONSIVE TO ABSCISIC ACID 28) (AtRAB28)	-1.1104694	0.25400083	-0.5115929	0.06278248
A0A1S2YEL8	O24456	Receptor for activated C kinase 1A	0.14733705	-0.1372275	0.10055817	0.07932811
A0A3Q7XNW1	P15455	12S seed storage protein CRA1 (Cruciferin 1) (AtCRU1)	0.39541937	0.53460938	0.65319235	-0.0333242
A0A1S2XSB9	P15456	12S seed storage protein CRB (Cruciferin 2) (AtCRU2) (Cruciferin B)	0.05409741	0.64853737	0.46826556	0.30989227
A0A1S2YSV6	P55034	26S proteasome non-ATPase regulatory subunit 4 homolog (26S proteasome regulatory subunit RPN10)	-0.1294573	0.00325757	-0.1679804	0.17997582
A0A1S2YBZ6	P06525	Alcohol dehydrogenase class-P (AtADH) (EC 1.1.1.1)	0.66751838	-0.0989213	0.15628652	-0.2346181
A0A1S2Z5Q8	O22809	NAD(P)-binding Rossmann-fold superfamily protein (Putative cinnamoyl-CoA reductase)	0.64319805	-0.5407374	0.02006562	-0.5428883
A0A1S2XHG2	O65282	20 kDa chaperonin, chloroplastic	0.18242918	0.29345062	-0.2196559	0.41489253
A0A1S2XT87	Q9LZR5	Histone deacetylase HDT3 (HD-tuins protein 3) (Histone deacetylase 2c)	-0.1146665	0.15945308	-0.3084035	0.0601576
A0A1S2XFZ1	Q9SIB9	Aconitate hydratase 3, mitochondrial (Aconitase 3) (mACO1) (EC 4.2.1.3)	0.37750017	-0.4632011	-0.0787552	-0.2222678
A0A1S2Z6X7	Q9LQ55	Dynamin-2B (EC 3.6.5.5) (Dynamin-like protein 3) (Dynamin-related protein 2B)	0.4288961	-0.1158241	0.28743817	-0.100542
A0A1S2Y7V6	P42759	Dehydrin ERD10 (Low-temperature-induced protein LTI45)	-1.2703345	0.99241522	-0.1204316	0.0034385

A0A1S2XBN2	Q06327	Linoleate 9S-lipoxygenase 1 (EC 1.13.11.58) (Lipoxygenase 1) (AtLOX1)	0.65015708	-0.1748523	0.29287441	-0.0137869
A0A1S2XP37	F4KCC2	E3 ubiquitin-protein ligase PRT6 (EC 2.3.2.27)	0.33687446	0.07351907	-0.1422096	0.27467158
A0A1S2YJU0	F4ISU2	Early endosome antigen	-0.8573443	0.30282356	-0.2634173	0.26646025
A0A1S2YB75	Q9ZVY7	Oil body-associated protein 1A	0.13637274	-0.9052055	0.29174459	-1.3620341
A0A1S2YNM8	Q944G9	Fructose-bisphosphate aldolase 2, chloroplastic (AtFBA2) (EC 4.1.2.13)	0.32220902	-0.3165247	-0.0134205	-0.042077
A0A1S2YHC3	F4INZ9	2-oxoglutarate (2OG) and Fe(II)-dependent oxygenase superfamily protein	0.55629509	-0.0549014	0.34812579	-0.1447743
A0A1S2Y255	F4J9A0	Nodulin/glutamine synthase-like protein	0.71158221	-0.0899257	0.21013946	0.32792684
A0A1S2Z4J7	P25696	Bifunctional enolase 2/transcriptional activator (EC 4.2.1.11) (	0.73433788	-0.1345641	0.37012791	-0.0508726
A0A1S2YRG3	Q9S7V4	HVA22-like protein a (AtHVA22a)	-0.3407742	-0.2926799	-0.1104765	-0.4939593
A0A1S2YH16	Q9FKB0	Sm-like protein LSM5 (AtLSM5)	-0.2162637	0.17356844	-0.0300793	-0.0275615
A0A3Q7XSJ1	Q8LDQ4	PP2A regulatory subunit TAP46 (2A phosphatase-associated protein of 46 kDa)	-0.1101781	-0.0634533	0.01107016	-0.1369727
A0A1S2XAC0	O81439	Probable plastid-lipid-associated protein 1, chloroplastic (AtPap1)	0.07774395	0.16172706	0.06160293	-0.1851125
B5LMK8	O03042	Ribulose bisphosphate carboxylase large chain (RuBisCO large subunit) (EC 4.1.1.39)	0.11748336	-0.2320174	-0.1232102	-0.0686661
A0A1S2YL17	Q93ZG7	DEAD-box ATP-dependent RNA helicase 38 (EC 3.6.4.13)	0.27212411	-0.1178702	-0.0368855	-0.2187376
A0A1S2XYS8	Q9LIL3	Aluminum induced protein with YGL and LRDR motifs (Uncharacterized protein At3g22850)	-0.2931389	-0.1685353	-0.2649778	-0.0301045
A0A1S3DW19	A0A1P8B3K6	CAP160 protein	-1.1458625	-0.0435648	-0.8169424	-0.1565535
A0A1S3E3Z2	F4KG52	CAP160 protein	-1.3552196	0.25456099	-0.4416017	0.06225122
