# Supplementary material for: Proteometabolomic Analysis Reveals Molecular Features Associated with Grain Size and Antioxidant Properties amongst Chickpea (Cicer arietinum L.) Seeds Genotypes
Source: Antioxidants (Basel). 2022 Sep 20;11(10):1850. doi: 10.3390/antiox11101850 (PMC9598084; doi:10.3390/antiox11101850)
Supplement: Supplementary file 1 [file antioxidants-11-01850-s001.zip › Table S4.pdf]

**Table S4.** Putative identification of core compounds detected in chickpea grains

| RT | m/z | Compound | Adduct | Adduct m/z | Error | Chemical group |
|---|---|---|---|---|---|---|
| | | | **Carbohydrates** | | | |
| 0.43 | 391.1015 | Galactopinitol | $[M+Cl]^-$ | 391.1013 | 0.5 | Hexose derivative |
| 0.46 | 563.1846 | Xylopyranosyl-arabinose | $[M-H]^-$ | 563.1829 | 3.0 | Oligosaccharide |
| 0.49 | 503.1636 | Oligosaccharide | $[M-H]^-$ | 503.1618 | 3.6 | Oligosaccharide |
| 1.84 | 863.4269 | Amylopectin | $[M+Cl]^-$ | 863.2441 | 3.2 | Polysaccharide |
| | | | **Organic acids** | | | |
| 0.71 | 191.0196 | Citric acid | $[M-H]^-$ | 191.0197 | -0.5 | Organic acid |
| | | | **Nitrogen Containing Compounds** | | | |
| 1.70 | 309.1088 | Glutamyltyrosine | $[M-H]^-$ | 309.1092 | -1.3 | Amino acid derivative |
| 2.28 | 203.0822 | Tryptophan | $[M-H]^-$ | 203.0826 | -2.0 | Amino acid |
| 2.81 | 293.1154 | Glutamylphenylalanine | $[M-H]^-$ | 293.1143 | 3.8 | Amino acid derivative |
| | | | **Phenolics** | | | |
| 0.48 | 381.0972 | Genistein-rhamnoside | $[M-2H_2O+H]^+$ | 381.0980 | -2.1 | Isoflavonoid glucoside |
| 0.49 | 711.2227 | Trihydroxy-heptamethoxy-biflavan | $[M+Cl]^-$ | 711.2214 | 1.8 | Biflavonoid |
| 1.72 | 315.0721 | Protocatechuic acid glucoside | $[M-H]^-$ | 315.0722 | -0.3 | Dihydrobenzoic acid glucoside |
| 2.27 | 188.0750 | Pentamethoxyflavanone | $[M+2H]^{2+}$ | 188.0756 | -3.2 | Polymethoxyflavone |
| | | | **Terpenoids** | | | |
| 1.72 | 311.1304 | Gibberellin | $[M-2H_2O+H]^+$ | 311.1289 | 4.8 | Diterpenoid |
| 2.80 | 295.1340 | Gibberellin A5 | $[M-2H_2O+H]^+$ | 295.1340 | 0.0 | Diterpenoid |
| 3.47 | 281.1389 | Dihydrophaseic acid | $[M-H]^-$ | 281.1394 | -1.8 | Sesquiterpenoid |
| 9.85 | 941.5111 | Soyasaponin I | $[M-H]^-$ | 941.5115 | -0.4 | Triterpenoid saponin |
| 10.09 | 795.4521 | Soyasaponin III | $[M-H]^-$ | 795.4536 | -1.9 | Triterpenoid saponin |
| | | | **Lipids** | | | |
| 12.93 | 478.2926 | Lysophospholipid | $[M+H]^+$ | 478.2928 | -0.4 | Lysophospholipid |
| 13.00 | 520.3401 | Lysolecithin | $[M+H]^+$ | 520.3398 | 0.6 | Phosphatidylcholine derivative |
| 13.26 | 478.2927 | Lysophospholipid | $[M+H]^+$ | 478.2928 | -0.2 | Lysophospholipid |
| 13.32 | 520.3400 | Lysolecithin | $[M+H]^+$ | 520.3398 | 0.4 | Phosphatidylcholine derivative |
| 13.38 | 313.2743 | Glycerolipid | $[M-H_2O+H]^+$ | 313.2743 | 0.0 | Glycerolipid |
| 13.60 | 496.3402 | Lysolecithin | $[M+H]^+$ | 496.3398 | 0.8 | Phosphatidylcholine derivative |
| 13.92 | 454.2928 | Lysophospholipid | $[M+H]^+$ | 454.2928 | 0.0 | Lysophospholipid |
| 13.99 | 496.3402 | Lysolecithin | $[M+H]^+$ | 496.3398 | 0.8 | Phosphatidylcholine derivative |
| 14.09 | 522.3556 | Lysolecithin | $[M+H]^+$ | 522.3554 | 0.4 | Phosphatidylcholine derivative |
| 14.38 | 480.3084 | Lysophospholipid | $[M+H]^+$ | 480.3085 | -0.2 | Lysophospholipid |
| 14.45 | 522.3562 | Lysolecithin | $[M+H]^+$ | 522.3554 | 1.5 | Phosphatidylcholine derivative |

RT: Retention time in minutes; m/z in Daltons; Error in ppm.
