# Supplementary material for: Proteometabolomic Analysis Reveals Molecular Features Associated with Grain Size and Antioxidant Properties amongst Chickpea (Cicer arietinum L.) Seeds Genotypes
Source: Antioxidants (Basel). 2022 Sep 20;11(10):1850. doi: 10.3390/antiox11101850 (PMC9598084; doi:10.3390/antiox11101850)
Supplement: Supplementary file 1 [file antioxidants-11-01850-s001.zip › Table S5.pdf]

Table S5: Endogenous content of phenolic compounds in chickpea grains.

Phenolic compounds	Samples				
	**SmallF1**	**BigF2**	**SmallCo**	**BigCo**	**Jumbo**
**Precursor**					
L-Phenylalanine	25.33 + 0.83	14.62 + 0.17	19.88 + 0.13	11.09 + 0.13	16.62 + 0.13
**Phenolic acids**					
4-Hydroxyphenylacetic acid	41.42 + 0.84	52.95 + 0.85	4.96 + 0.15	39.65 + 0.33	52.94 + 0.63
4-Hydroxybenzoic acid	14.26 + 0.13	6.53 + 0.22	6.35 + 0.57	2.50 + 0.07	4.60 + 0.05
Gentisic acid	9.74 + 0.06	7.85 + 0.50	14.23 + 1.80	4.03 + 0.08	5.39 + 0.06
Protocatechuic acid	0.44 + 0.04*	0.28 + 0.02*	0.22 + 0.01*	0.21 + 0.00*	0.30 + 0.02*
Vanillic acid	0.36 + 0.02*	0.09 + 0.01*	0.07 + 0.00*	0.01 + 0.00*	0.06 + 0.01*
Sinapic acid	0.33 + 0.01*	0.27 + 0.01*	0.33 + 0.01*	0.15 + 0.02*	0.18 + 0.01*
Salicylic acid	0.30 + 0.00*	0.06 + 0.01*	0.16 + 0.01*	---	---
4-Coumaric acid	0.20 + 0.02*	0.03 + 0.01*	0.07 + 0.01*	---	0.03 + 0.01*
Ferulic acid	0.14 + 0.02*	---	---	---	---
**Phenolic aldehyde**					
Vanillin	0.17 + 0.00*	0.12 + 0.01*	0.05 + 0.04*	0.05 + 0.01*	---
**Flavonoid**					
Kaempferol-3-O-glucoside	0.96 + 0.02*	0.39 + 0.01*	0.42 + 0.01*	0.38 + 0.03*	0.19 + 0.01*
**Lignan**					
Secoisolariciresinol	1.18 + 0.08*	1.90 + 0.13*	1.54 + 0.18*	1.00 + 0.04*	---

Concentration is expressed in µg/g of dried methanolic extract. It is shown the average and standard deviation of three determinations. Value determined below the limit of quantification (*). Phenolic compound not identified (---).
