# Supplementary material for: Proteometabolomic Analysis Reveals Molecular Features Associated with Grain Size and Antioxidant Properties amongst Chickpea (Cicer arietinum L.) Seeds Genotypes
Source: Antioxidants (Basel). 2022 Sep 20;11(10):1850. doi: 10.3390/antiox11101850 (PMC9598084; doi:10.3390/antiox11101850)
Supplement: Supplementary file 1 [file antioxidants-11-01850-s001.zip › Table S6.pdf]

Table S6. Conditions for each compound used in MRM (Multiple Reaction Monitoring)

Compound	dMRM Transition			Mass Spectrometric Conditions			Quantification Conditions		
	Precursor Ion	Product Ion	RT	Collision Energy	Fragmentor	Polarity	Quantification Range (μM)	Regression Type	R^2
Shikimic acid	173.1	111.1	0.49	10	100	Negative	0.5–19	Quadratic	0.99
Gallic acid	169.0	125.2	1.4	10	100	Negative	1–19	Quadratic	0.99
L-Phenylalanine	166.1	131.0	1.92	10	100	Positive	0.25–19	Quadratic	0.99
Protocatechuic acid	153.0	109.1	2.5	10	100	Negative	0.25–19	Quadratic	0.99
4-Hydroxybenzoic acid	137.1	92.8	3.76	10	100	Negative	0.25–19	Quadratic	0.99
Gentisic acid	153.0	109.0	3.83	10	100	Negative	0.25–19	Quadratic	0.99
4-Hydroxyphenylacetic acid	107.1	77.0	4.72	20	140	Positive	0.25–19	Quadratic	0.99
(-)-Epigallocatechin	305.1	125.0	4.83	20	140	Negative	1–17	Quadratic	0.99
(+)-Catechin	291.0	138.9	5.07	10	100	Positive	0.5–19	Quadratic	0.99
Vanillic acid	169.0	93.0	5.12	10	100	Positive	0.25–19	Quadratic	0.99
Scopolin	355.1	193.0	5.25	20	100	Positive	0.25–19	Quadratic	0.99
Chlorogenic acid	355.1	163.0	5.34	10	100	Positive	0.25–19	Quadratic	0.99
Caffeic acid	181.0	163.	5.38	10	100	Positive	0.5–19	Quadratic	0.99
Malvin	655.1	331.1	5.82	40	100	Positive	0.5–19	Quadratic	0.99
Kuromanin	449.0	286.9	6.34	30	100	Positive	0.5–19	Quadratic	0.99
Procyanidin B2	577.1	425.1	6.4	10	100	Negative	1–19	Quadratic	0.99
Vanillin	153.0	124.9	6.52	10	100	Positive	0.25–19	Quadratic	0.99
Keracyanin	595.2	287.1	6.88	20	100	Positive	0.5–19	Quadratic	0.99
(−)-Epicatechin	291.0	138.8	6.96	10	100	Positive	0.5–19	Quadratic	0.99
4-Coumaric acid	165.0	147.0	7.21	10	100	Positive	0.25–19	Quadratic	0.99
Mangiferin	423.0	302.8	7.32	10	100	Positive	0.5–19	Quadratic	0.99
Umbelliferone	163.0	107.0	7.64	30	100	Positive	0.25–19	Quadratic	0.99
(−)-Gallocatechin gallate	458.9	139.0	7.95	20	80	Positive	1–19	Quadratic	0.99
Scopoletin	193.0	133.0	8.4	10	100	Positive	0.25–19	Quadratic	0.99
Ferulic acid	195.1	145.0	8.6	20	100	Positive	0.25–19	Quadratic	0.99
Quercetin 3,4-di-*O*-glucoside	627.0	302.9	8.77	10	100	Positive	0.5–19	Quadratic	0.99
3-Coumaric acid	165.05	147.04	8.81	10	100	Positive	0.5–19	Quadratic	0.99
Salicylic acid	137.0	93	9.15	10	100	Negative	0.5–19	Quadratic	0.99
Sinapic acid	225.1	207.1	9.16	10	100	Positive	0.25–19	Quadratic	0.99
Epicatechin gallate	443.1	123.0	9.83	10	100	Positive	1–19	Quadratic	0.99
Ellagic acid	300.5	145.0	9.98	30	170	Negative	1–19	Quadratic	0.99
Myricitrin	465.0	318.9	10.03	10	100	Positive	1–19	Quadratic	0.99
Pelargonidin	271.1	121	10.22	20	10	Positive	1–19	Quadratic	0.97
Quercetin 3-D-galactoside	465.0	302.9	10.26	10	100	Positive	0.25–19	Quadratic	0.99
Rutin	611.0	302.9	10.35	10	100	Positive	0.25–19	Quadratic	0.99
p-Anisic acid	153.1	109.0	10.45	5	120	Positive	0.25–19	Quadratic	0.99
Quercetin 3-glucoside	465.0	303.0	10.57	10	100	Positive	0.25–19	Quadratic	0.99
Luteolin 7-*O*-glucoside	449.0	287.0	10.77	10	100	Positive	0.5–19	Quadratic	0.99
Malvidin	331.1	287.1	11.14	20	100	Positive	1–17	Quadratic	0.96
2,4-Dimethoxy-6-methylbenzoic acid	197.0	179.0	11.41	5	80	Positive	0.25–19	Quadratic	0.99
Penta-*O*-galloyl-B-D-glucose	771.1	153.0	11.68	20	100	Positive	0.5–19	Quadratic	0.99
Kaemperol 3-*O*-glucoside	449.0	286.9	11.91	10	100	Positive	0.25–19	Quadratic	0.99
Quercitrin	449.1	303.1	11.95	10	100	Positive	0.5–19	Quadratic	0.99
Naringin	273.0	153.0	12.13	10	120	Positive	0.25–19	Quadratic	0.99
Myricetin	317.0	179.0	12.29	10	100	Negative	0.5–15	Quadratic	0.99
Hesperidin	609.1	301.1	12.68	20	100	Negative	0.5–19	Quadratic	0.99
trans-Resveratrol	229.1	135.0	12.69	10	100	Positive	0.5–19	Quadratic	0.99
Rosmarinic acid	361.1	163.0	12.8	10	100	Positive	0.5–19	Quadratic	0.99

Secoisolariciresinol	363.2	137.1	13.02	20	100	Positive	0.5–19	Quadratic	0.99
Phloridzin	435.0	272.9	13.04	10	100	Negative	0.25–19	Quadratic	0.99
trans-Cinnamic acid	149.1	131.0	14.08	10	100	Positive	0.25–19	Quadratic	0.99
Psoralen	187.0	131.1	14.99	20	100	Positive	0.25–19	Quadratic	0.99
Quercetin	302.9	153.1	15.18	35	100	Positive	1–19	Quadratic	0.99
Luteolin	287.1	153.0	15.28	30	100	Positive	0.5–19	Quadratic	0.99
Angelicin	187.0	131.1	15.75	20	100	Positive	0.5–19	Quadratic	0.99
Naringenin	271.0	151	16.79	10	100	Negative	0.5–19	Quadratic	0.99
Apigenin	271.0	153.0	17.45	30	100	Positive	0.5–19	Quadratic	0.99
Matairesinol	359.2	137.1	17.55	10	100	Positive	0.25–19	Quadratic	0.99
Kaempferol	287.1	153.0	17.81	30	100	Positive	0.25–19	Quadratic	0.99
Hesperetin	303.1	177.1	18.06	20	100	Positive	0.25–19	Quadratic	0.99
Podophyllotoxin	415.1	397.1	19.01	10	100	Positive	0.25–19	Quadratic	0.99
Methyl cinnamate	163.1	131.0	21.46	6	100	Positive	0.25–1	Quadratic	0.99
Nordihydroguaiaretic acid	303.0	193.1	22.72	10	100	Positive	0.5–19	Quadratic	0.99
Chrysin	255.1	153.0	22.89	40	100	Positive	0.25–19	Quadratic	0.99
Kaempferide	301.0	258.2	24.38	20	100	Positive	0.5–19	Quadratic	0.99
Emodin	269.0	225.0	27.45	20	150	Negative	1–17	Quadratic	0.99
Chrysophanol	255.1	153.0	31.34	40	100	Positive	0.25–19	Quadratic	0.99

RT: Retention time in minutes; m/z in Daltons; Collision energy and Fragmentor values are in Volts.
