# Supplementary figures and images for: Proteometabolomic Analysis Reveals Molecular Features Associated with Grain Size and Antioxidant Properties amongst Chickpea (Cicer arietinum L.) Seeds Genotypes
Source: Antioxidants (Basel). 2022 Sep 20;11(10):1850. doi: 10.3390/antiox11101850 (PMC9598084; doi:10.3390/antiox11101850)

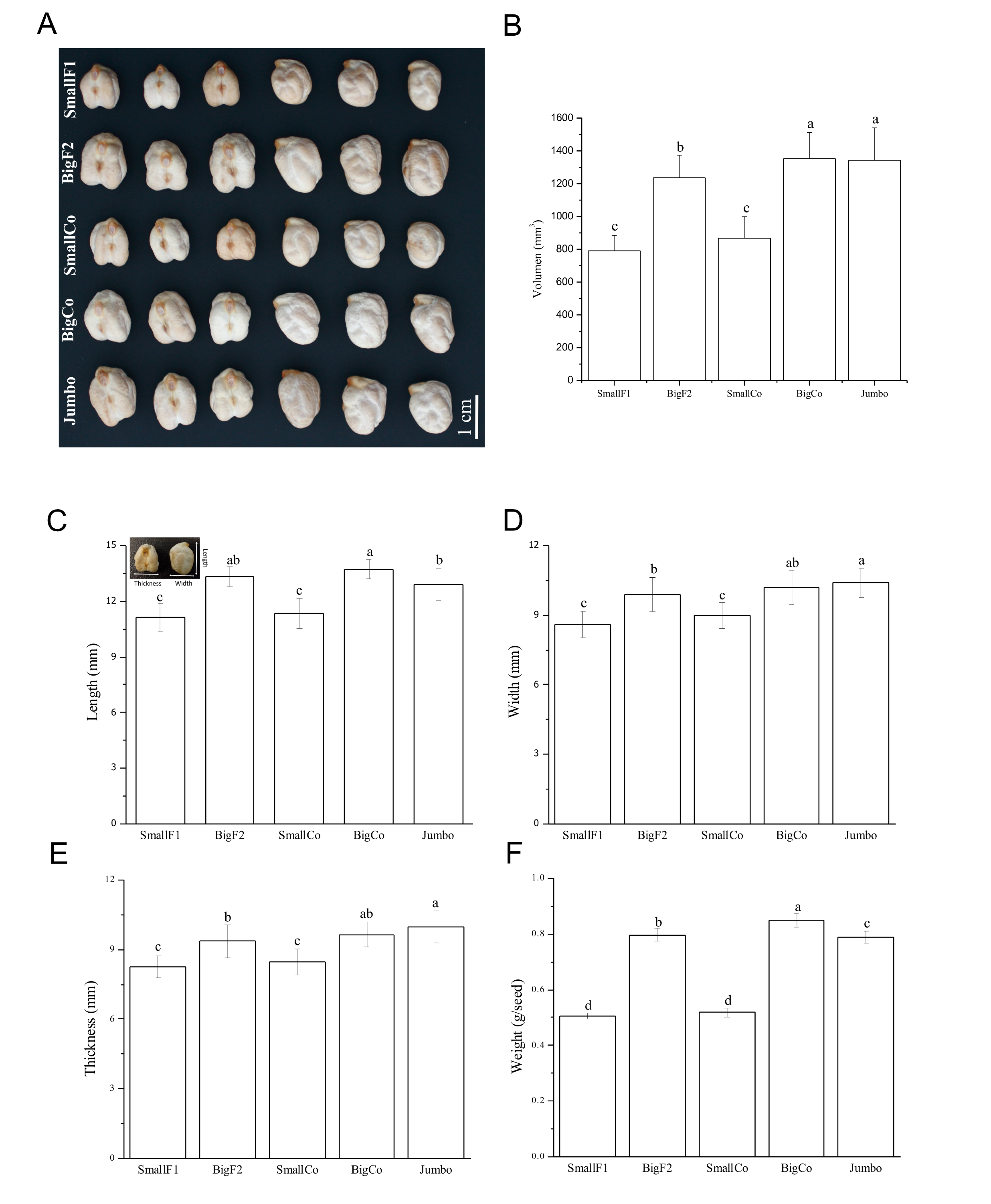

Supplement: Supplementary file 1 [file antioxidants-11-01850-s001.zip › Figure S1.tif]

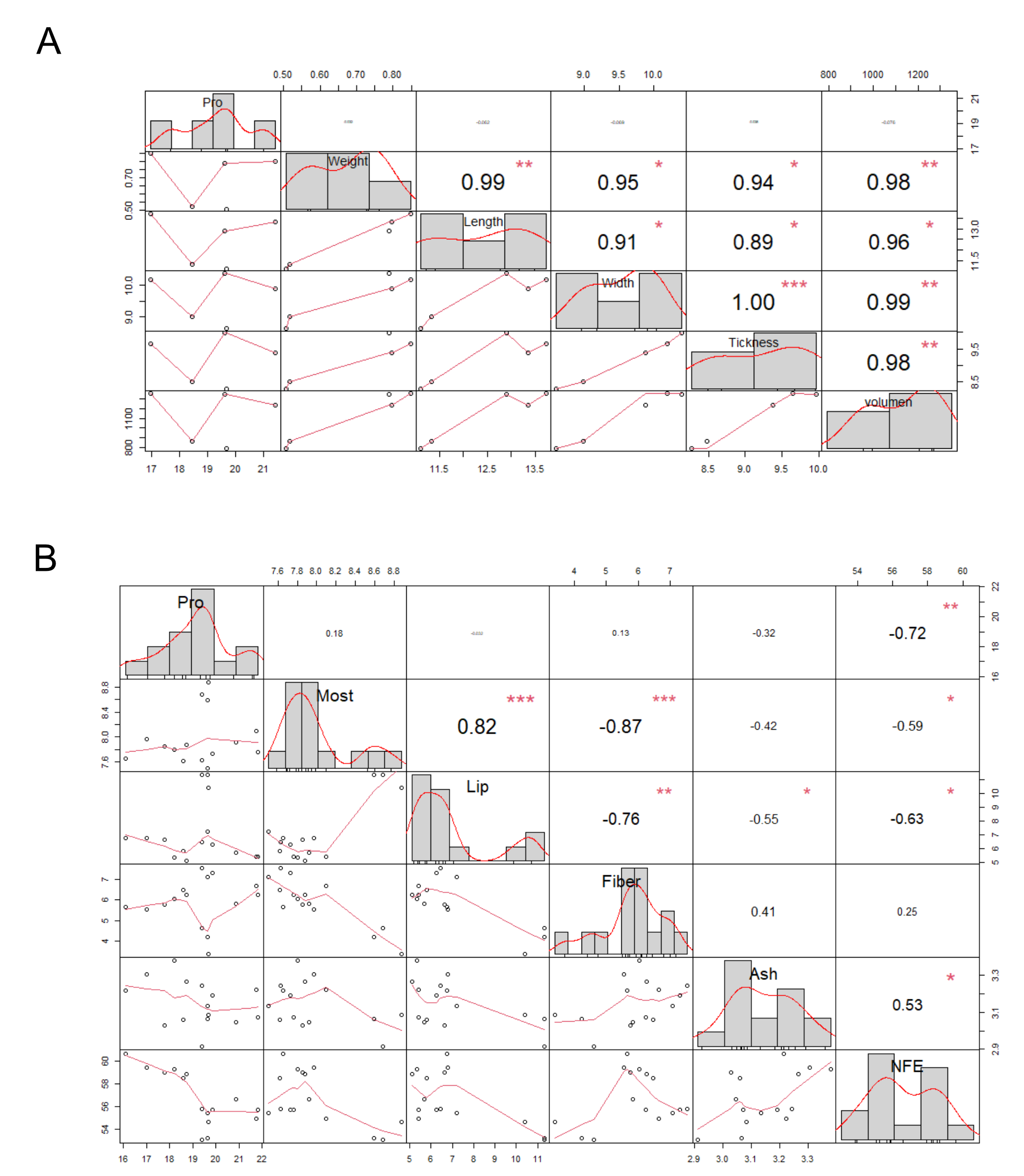

Supplement: Supplementary file 1 [file antioxidants-11-01850-s001.zip › Figure S2.tif]
